# Genome Sequencing of the Pyruvate-producing Strain *Candida glabrata* CCTCC M202019 and Genomic Comparison with Strain CBS138

**DOI:** 10.1038/srep34893

**Published:** 2016-10-07

**Authors:** Nan Xu, Chao Ye, Xiulai Chen, Jia Liu, Liming Liu, Jian Chen

**Affiliations:** 1 State Key Laboratory of Food Science and Technology, Jiangnan University, 1800 Lihu Road, Wuxi, Jiangsu 214122, China; 2 Key Laboratory of Industrial Biotechnology, Ministry of Education, School of Biotechnology, Jiangnan University, 1800 Lihu Road, Wuxi, Jiangsu 214122, China

## Abstract

*Candida glabrata* CCTCC M202019 as an industrial yeast strain that is widely used to produce α-oxocarboxylic acid. Strain M202019 has been proven to have a higher pyruvate-producing capacity than the reference strain CBS138. To characterize the genotype of the M202019 strain, we generated a draft sequence of its genome, which has a size of 12.1 Mbp and a GC content of 38.47%. Evidence accumulated during genome annotation suggests that strain M202019 has strong capacities for glucose transport and pyruvate biosynthesis, defects in pyruvate catabolism, as well as variations in genes involved in nutrient and dicarboxylic acid transport, oxidative phosphorylation, and other relevant aspects of carbon metabolism, which might promote pyruvate accumulation. In addition to differences in its central carbon metabolism, a genomic analysis revealed genetic differences in adhesion metabolism. Forty-nine adhesin-like proteins of strain M202019 were identified classified into seven subfamilies. Decreased amounts of adhesive proteins, and deletions or changes of low-complexity repeats and functional domains might lead to lower adhesion and reduced pathogenicity. Further virulence experiments validated the biological safety of strain M202019. Analysis of the *C. glabrata* CCTCC M202019 genome sequence provides useful insights into its genetic context, physical characteristics, and potential metabolic capacity.

*Candida glabrata*, previously designated *Torulopsis glabrata*, is a haploid yeast belonging to the Saccharomycetaceae family and the *Candida* genus. The *C. glabrata* CCTCC M202019 strain, originally screened from fertile soil niches, endured long-term and repeated domestication in laboratory broth^1^,^2^, and when used on an industrial scale, it can accumulate 84.2 g·L^−1^ pyruvate^3^. Pyruvate is an important chemical moiety that has been widely used in chemosynthesis, agrochemistry, dietary supplements, and pharmaceutical applications^4^,^5^. In addition, strain M202019 has the potential to produce α-ketoglutarate^6^, fumarate^7^, acetoin^8^, malate^9^, and diacetyl^10^, as demonstrated by metabolic engineering experiments that increased the carbon flux from pyruvate nodes into these downstream chemicals. However, there is lack of a systematic understanding of its high pyruvate production capacity. To gain a comprehensive understanding of the genetic characteristics of strain M202019 for industrial-scale pyruvate production, and to improve its exploitation in industrial biotechnology, the genome of *C. glabrata* CCTCC M202019 needs to be sequenced, compared, and analyzed.

CBS138 was the first *C. glabrata* strain to have its whole genome sequenced^11^; *C. glabrata* is the second most frequent opportunistic yeast pathogen to be isolated from human feces and mucosa^12^. *C. glabrata* CBS138 can cause urogenital tract and bloodstream infections as a result of its own virulence-related traits, such as adherence, drug resistance, pigment production, acidic tolerance, and *ace2* mutations^13^. Among them, adherence is thought to be an extremely important virulence factor, and adherence is mediated by lectins that are encoded by epithelial adhesin (EPA) genes^14^. These EPA genes encode glycosylphosphatidylinositol (GPI)-anchored cell wall proteins that are covalently bound to cell wall glycoconjugates^15^. Its special cell wall architecture may help *C. glabrata* CBS138 to adhere to biotic and abiotic surfaces of mammalian cells. Both the niches and evolutionary history of strain CBS138 different from those of *C. glabrata* CCTCC M202019, which might lead to differences in the physiological and metabolic performance of these strains. The currently available *C. glabrata* CBS138 genome is a valuable tool for functional and comparative genomic research of *C. glabrata*.

In this study, we first showed that *C. glabrata* CCTCC M202019 has a higher pyruvate-producing capacity than *C. glabrata* CBS138. To determine key genotypic properties, the whole genome of *C. glabrata* CCTCC M202019 was *de novo* sequenced and mapped using high-throughput sequencing technology. The comprehensiveness and reliability of the function of M202019 genes were ensured by using various prediction and annotation tools. Then, a comparative genomic analysis was performed between the two strains. They shared highly similar genome structure and gene-order features, and genetic differences mainly appeared in pyruvate-related central carbon metabolism and toxic metabolism. Furthermore, the mechanism underlying the decreased virulence of *C. glabrata* CCTCC M202019 was resolved and validated. This study not only provides a genomic platform to understand the physiological and metabolic mechanisms of *C. glabrata*, but it also identifies potential genetic targets for the optimization of carbon metabolism and the reduction of toxicity.

## Results and Discussion

### Pyruvate fermentation in *C. glabrata* CCTCC M202019 and CBS138

**Figure 1:**
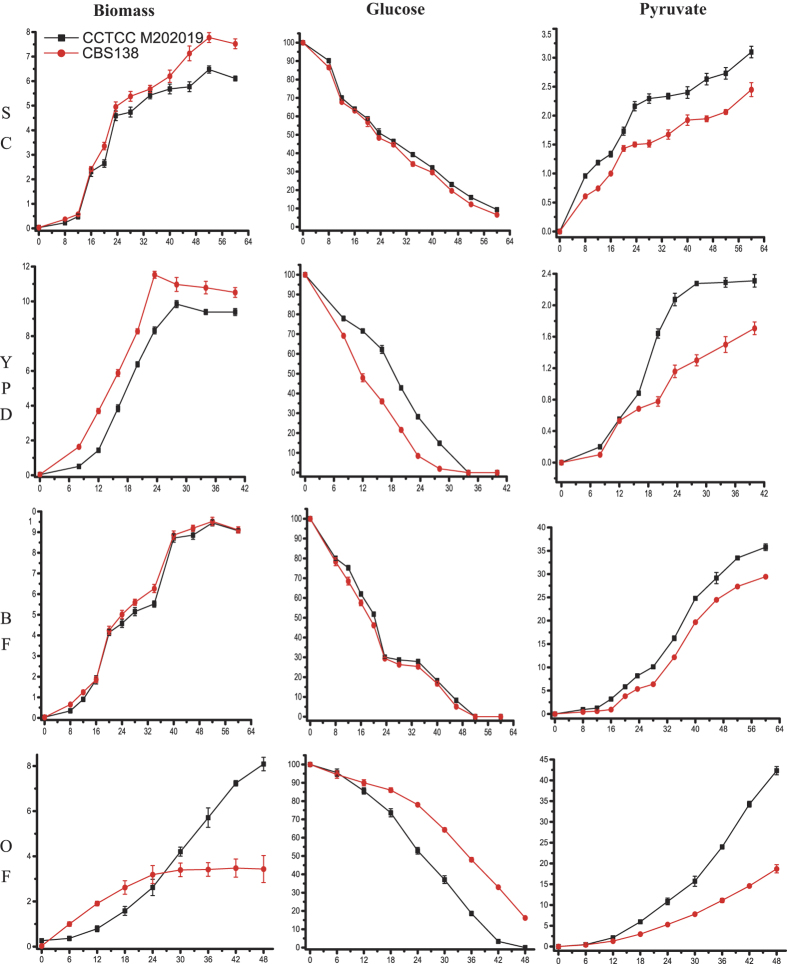
Pyruvate fermentation process of *C. glabrata* CCTCC M202019 and CBS138. Synthetic complete medium (SC), yeast extract peptone dextrose medium (YPD), basal fermentation medium (BF), and optimized fermentation medium (OF); units, g·L^−1^.

It was reported that pyruvate production by *C. glabrata* CCTCC M202019 has reached an industrial level, and that other *C. glabrata* strains can accumulate pyruvate^16^. However, the pyruvate production capacity of *C. glabrata* CBS138 was never investigated. Therefore, pyruvate production by *C. glabrata* CCTCC M202019 and CBS138 was analyzed (Fig. 1). In commonly used media, i.e., synthetic complete (SC), yeast extract peptone dextrose (YPD), and basal fermentation (BF) media, strain CBS138 grew slightly faster than strain M202019, especially in YPD medium (Fig. 1 and Supplementary Table S1a–c). An obvious growth difference was observed in an optimized fermentation (OF) medium. After culturing for 48 h, strain M202019 reached 8.09 g·L^−1^ of biomass and just entered into stationary phase, whereas the biomass of strain CBS138 only reached 3.44 g·L^−1^ (Supplementary Table S1d), and it entered into stationary phase after approximately 30 h. Glucose consumption of the two strains was similar to their cell growth curves in these media (Fig. 1 and Supplementary Table S1a–c). Glucose was consumed slightly faster by strain CBS138, compared with strain M202019, especially in YPD medium. In contrast, in OF medium, the glucose consumption rates of strains M202019 and CBS138 were 2.03 g·L^−1^·h^−1^ and 1.74 g·L^−1^·h^−1^, respectively. Additionally, pyruvate production by *C. glabrata* CCTCC M202019 was 39%, 61%, 66%, and 95% higher than that of strain CBD138 in SC, YPD, BF, and OF media, respectively. The most significantly improvement in OF could be reflected by the fact that 42.32 g·L^−1^ pyruvate accumulated in strain M202019, while strain CBS138 only accumulated 18.7 g·L^−1^ pyruvate at 48 h. Meanwhile, pyruvate yields and productivity by strain M202019 were 0.21 g·g^−1^ and 0.41 g·L^−1^·h^−1^ higher, respectively, than those in strain CBS138 (Supplementary Table S1d). In general, although the two strains accumulated pyruvate, either in commonly used media or in an optimized medium, the pyruvate production, yield, and productivity of strain M202019 were higher than those of strain CBS138, especially in the optimized medium. The greater pyruvate productivity exhibited by *C. glabrata* CCTCC M202019 might have evolved selectively during laboratory mutagenesis. To extensively analyze phenotypical difference and explore candidates for improving the pyruvate production capacity, the whole genome of the pyruvate-producing M202019 strain was sequenced and compared with the genome of strain CBS138.

### Genome sequencing and genome characteristics of *C. glabrata* CCTCC M202019

The *C. glabrata* CCTCC M202019 genome was sequenced using the Illumina Solexa HiSeq 2000 platform. The 12.1 Mbp genome sequence, with an average G + C content of 38.47%, was acquired from a 150 bp pair-end library with 208-fold coverage, a 300 bp pair-end library with 165-fold coverage, and one mate-pair library with 106 fold coverage (Supplementary Fig. S1). High-quality reads were assembled into 111 contigs and 74 scaffolds. The N50 sizes of the contigs and scaffolds were 659,495 and 775,409, respectively (Table 1). A total of 5345 genes were predicted, 293 of which were generated by alternative splicing. Subsequently, gene function was annotated, including 4788 genes that were classified by Gene Ontology and 3088 genes that were classified by EuKaryotic Orthologous Groups (KOG) (Supplementary Dataset S1). In addition, 191 tRNA and six rRNA genes were identified (Table 1). The total repeat sequences occupied 1.15% of the genome (Supplementary Table S2), which included 16 short interspersed elements (1673 bp), 11 unclassified interspersed elements (11,449 bp), 2644 simple repeats (121,642 bp), 365 low-complexity repeats (17,220 bp), and two long terminal repeats (776 bp).

### Overview of comparative genomics between *C. glabrata* CCTCC M202019 and CBS138

Comparative genomics of *C. glabrata* CCTCC M202019 and CBS138 was investigated using the GO and KOG functional annotations, a homologous comparison of gene sequences, and the detection of single nucleotide polymorphisms (SNPs). First, 2792 GO terms were shared by the two strains (Supplementary Dataset S1), while strains M202019 and CBS138 had 37 and 24 unique GO terms, respectively (Supplementary Table S3). The two strains shared 2301 KOG categories (Supplementary Dataset S1), while 188 and 63 unique KOG categories were found in the M202019 and CBS138 strains, respectively (Supplementary Fig. S2). Additionally, the same genes of strains M202019 and CBS138 occupied 94.6% and 95.1% (Supplementary Dataset S1), respectively, of their genomes, which accounted for the majority of the core metabolic pathways. Other genes were ranked by their identities (Supplementary Table S4). The total of 275 and 159 genes exhibited greater than 90% identity in the M202019 and CBS138 strains, respectively. Sixteen and 43 genes exhibited less than 90% identity in the M202019 and CBS138 strains. Noticeably, 74.3% of them were predicted to function in cell wall adhesion. Twelve unique genes in strain CBS138 belonged to adhesion clusters and genetic information process. The unique gene in strain M202019 encoded a ribulose-1,5-bisphosphate carboxylase/oxygenase subunit. A total of 205 SNPs were preliminarily identified (Supplementary Table S5), 61 of which were scattered in the regulatory regions of 25 genes (Supplementary Table S7), while the rest were located in the open reading frames of 51 genes (Supplementary Table S6). Among the 51 genes, 35 belonged to verified or hypothetical proteins, and 16 were pseudogenes. The 35 verified or hypothetical proteins functioned in cell adhesion (22.9%), central carbon metabolism (22.9%), and non-metabolic cellular processes (45.7%), and 31 of them acquired sense mutations, while the other four contained nonsense mutations. Additionally, 13 of the 16 pseudogenes were predicted to be related to cell adhesion. Overall, the comparative genomics analysis suggests a high similarity of these two closely related strains, and the limited number of genetic mutations is likely related to certain phenotypic differences.

### Genomic characteristics accounting fhor high pyruvate productivity in *C. glabrata* CCTCC M202019

The main factors influencing pyruvate metabolism in *C. glabrata* include pyruvate biosynthesis from glucose via glycolysis, the pentose phosphate shunt, and methylglyoxal degradation pathways, and an efficient uptake and transport system that is important for pyruvate production^17^. Additionally, the high pyruvate accumulation could result from the auxotrophies of four vitamins, which function as the cofactors of key enzymes in pyruvate metabolic pathways that weaken pyruvate degradation^18^. Furthermore, pyruvate production can be enhanced by altering the transmission of reducing power, as well as energy production, by engineering oxidative phosphorylation to strengthen NADH oxidation^19^ and decrease ATP levels^20^. Finally, some other by-products, such as tricarboxylic acid metabolites, could compete with pyruvate for carbon flux. Compared to the genome of *C. glabrata* CBS138, the genome of strain M202019 had some changes in central carbon metabolism (Table 2), including transport proteins such as glucose transporters, niacin transporters, an acetic acid transporter, and a transporter that transfers dicarboxylic acid transporter between mitochondria and the cytoplasm. Other genes were involved in oxidative phosphorylation, including cytochrome C oxidase, cytochrome C reductase, an F-type ATPase, and assembly and biogenesis proteins of electron transport chain complex IV. Other genes that are relevant to metabolism include acetyl-CoA hydrolase, and involved in acetate formation and glutamate synthase as joints of carbon and nitrogen metabolism. The aforementioned changes in central carbon metabolism genes might be associated with higher levels of pyruvate production by *C. glabrata* CCTCC M202019.

### Identification and classification of adhesin proteins in *C. glabrata* CCTCC M202019

**Figure 2:**
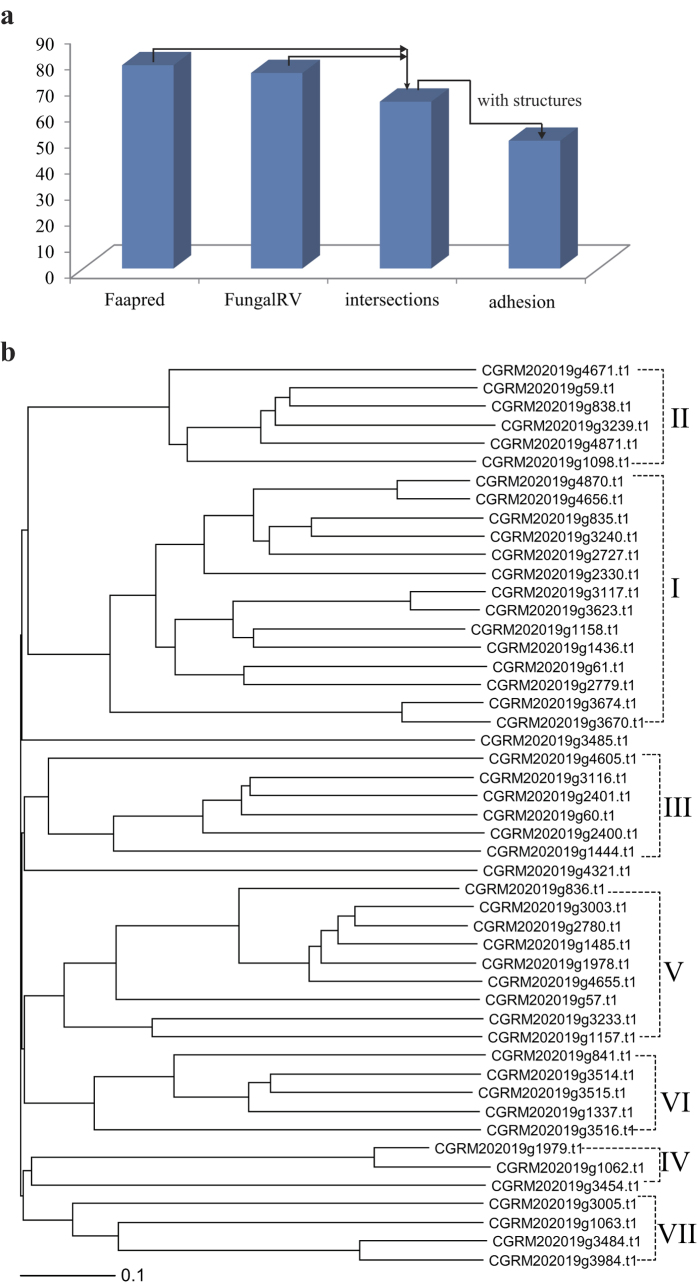
Identification and classification of adhesin-like proteins in *C. glabrata* CCTCC M202019 (**a**) Identification of adhesin-like proteins (**b**) Classification of adhesin-like proteins.

In addition to those involved pyruvate-related central carbon metabolism, many genetic differences were also identified in adhesion metabolism. In *C. glabrata* CBS 138, 62 adhesion proteins were predicted by FungalRV using a threshold of 0.511 including 20 adhesins identified in the proteome^21^. FaaPred also successfully predicted adhesin-like proteins and supplement-related annotations such as CAGL0I10246g, CAGL0J02508g, and CAGL0I11011g in *C. glabrata* CBS 138. The intersection of results from two programs were considered to reduce false positives. Using the protein sequences of *C. glabrata* CCTCC M202019, 75 and 78 fungal adhesins and adhesin-like proteins were predicted by FungalRV and FaaPred, respectively. Among the shared 64 proteins, 49 possessed typical adhesion protein structures (Fig. 2a), including a GPI-anchor, low-complexity serine (S)/threonine (T) repeats, conserved (V)SHITT/TTVVT amino acid motifs, hyphally-regulated cell wall proteins (Hyphal_reg_CWP), PA14 and its similar structural domain, and flocculin repeats (Supplementary Table S8). These 49 proteins are thought to be putative adhesins of strain M202019, and they were divided into seven subtypes according to their amino-terminal structures (Fig. 2b), which is consistent with adhesin protein classification for *C. glabrata* CBS138^22^. The largest subgroup (I) contained 14 proteins, and its nearest homology subgroup (II) comprised six proteins. Both subgroups had conserved PA14 and PA14-like domains, wherein g4656, g835, and g3240 also had Hyphal_reg_CWP and flocculin repeats. Three adhesins in subset IV had a GPI anchor. Almost all of the adhesins in subgroups V and VI contained Hyphal_reg_CWP protein structures, which indicated the functional similarities of the two subgroups. Non-characteristic domains were found in subgroups III and VII, while all seven families possessed low-complexity repeats. Additionally, the conserved repeats SHITT and TTVVT were distributed in most subgroups, including subgroups I, III, IV, and V.

### Structural variations of adhesins in *C. glabrata*

The comparative genomics analysis suggested that only six adhesins were shared by the two strains, and the remaining 54 hypothetical proteins and 12 pseudogenes in *C. glabrata* CBS138 all differed. For 47 of the 54 adhesins, variations located in low-complexity repeats were rich in serine and threonine. The mutations and deletions in the aforementioned tandem repeats could cause the amino-terminal effector domains of these adhesins to become buried in the cell wall^23^. Compared with low-complexity repeats, other structural domains seemed to exhibit lower variation frequencies, including the PA14 domains of CAGL0E06688g, the flocculin repeats of CAGL0I07293g and CAGL0I00220g, Hyphal_reg_CWP of CAGL0J02530g and CAGL0F09273g, and the GPI-anchor regions of CAGL0L09911g, CAGL0L00157g, and CAGL0H10626g. These functional domains are reported to be individually instrumental in adhesion to human tissue^14^,^24^,^25^. For example, PA14 domains, specifically the ligand-binding domains of EPA proteins, were found in bacterial toxins, glucosidases and adhesins. Deletion of *C. glabrata* genes with PA14 domains such as CAGL0I10098g reduce its adherence to endothelial cells^26^. Flocculin repeats involved in flocculation and cell adhesion, and yeast strains with a larger number of repeats in the *FLO1*(YAR050W) gene exhibit better adhesion than those with a smaller number of repeats^27^. Therefore, these changes in the functional domains of adhesins were proposed to reduce the adherence of *C. glabrata*. In addition, of the 12 changed pseudogenes that were associated with cell aggregation, the distribution of the mutation sites was comparatively dispersed in five pseudogenes, but relatively concentrated in the other seven pseudogenes (Supplementary Table S9). These differences in adhesin-encoding genes might reduce cell adherence and virulence.

### Evaluation of the biological safety of *C. glabrata* CCTCC M202019

**Figure 3:**
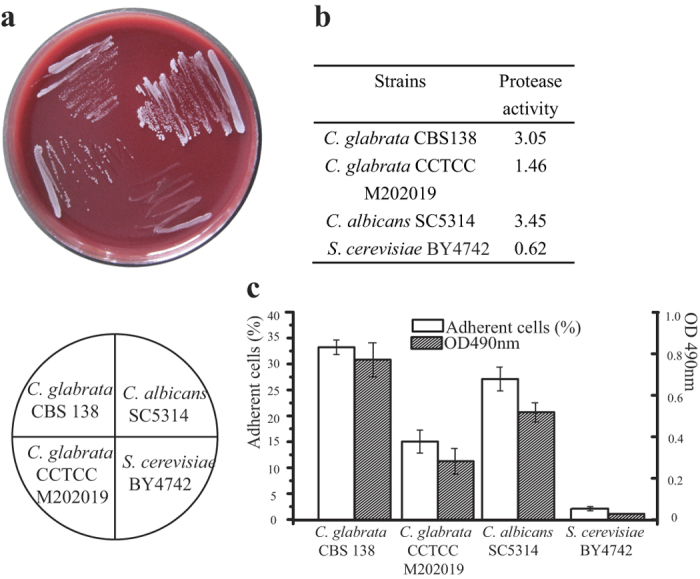
Virulence and adhesion testing of *C. albicans* SC5314, *S. cerevisiae* BY4742, and *C. glabrata* CCTCC M202019 and CBS138 (**a**) Cell growth on Columbia agar base; (**b**) protease activity; (**c**) adhesion to endothelial cells and 96-microwell plates.

The virulence and adherence of *C. glabrata* CCTCC M202019 was tested and compared to validate the aforementioned hypothesis. The pathogenicity of strains M202019 and CBS138 could be reflected in their ability to grow on Columbia agar base; thus, the growth of *Candida albicans* SC5314, *Saccharomyces cerevisiae*, and *C. glabrata* CCTCC M202019 and CBS138 was tested on this medium. The highly virulent *C. albicans* exhibited the highest growth rate, followed by strain CBS138 while strain M202019 and *S. cerevisiae* grew slowly (Fig. 3a). The virulence of such strains is generally proportional to their protease producing capacity. Among these four strains, *C. albicans* SC5314 and *C. glabrata* CBS138 exhibited a high protease-producing capacity, with protease activities of 3.45 and 3.05, respectively (Fig. 3b). Strain M202019 was a less virulent strain, with a protease activity of 1.46, while *S. cerevisiae* colonies did not generate transparent halos. Thus, based on its growth rate and protease-producing capacity, *C. glabrata* CCTCC M202019 appears to be less virulent and, hence, safer than *C. glabrata* CBS138.

The toxicity and pathogenicity of *Candida* mainly results from a membrane structure that enables adherence to the surfaces of biological or non-biological materials^28^. Here, the adhesion of *C. albicans* SC5314, *S. cerevisiae*, and *C. glabrata* CCTCC M202019 and CBS138 to endothelial cells and the inner wall of pipes was investigated. The adhesion rate of strain CBS138 to endothelial cells was 33%, and the next most adhesive strain was the highly virulent *C. albicans*. The adhesion rate of strain M202019 (15%) was less than half of that of *C. glabrata* CBS138. Almost no endothelial cells exhibited adherent *S. cerevisiae*, which is non-virulent. The adhesion of the four experimental strains on 96-microwell plates is also shown in Fig. 3c. *C. glabrata* CBS138 had the strongest adhesion, followed by *C. albicans* SC5314. Compared with *C. glabrata* CBS138, the adhesion of strain M202019 on 96-microwell plates was 63.6% lower. The absorbance value of adherent cells was close to 0 for *S. cerevisiae*. We conclude that the adhesive capacity of strain M202019 was only 40–50% of that of *C. glabrata* CBS138 on either biological (endothelial cells) or non-biological (96-microwell plates) surfaces. The lower adhesive ability of the pyruvate-producing strain *C. glabrata* CCTCC M202019 was predicted by the genomics analysis, possibly due to the decreased amounts of adhesive proteins, and deletion or mutations in low-complexity repeats in this strain.

## Methods

### Genome sequencing and annotation

*C. glabrata* CCTCC M202019 was preserved in the China Center for Type Culture Collection (Wuhan, Hubei, China). Genomic DNA was extracted from fresh cells during logarithmic growth phase, and DNA was sequenced using the Illumina (San Diego, CA, USA) Solexa HiSeq 2000 platform. Three libraries were constructed. Two paired-end libraries were prepared with 150 bp and 300 bp fragments. These libraries generated 22,645,461 reads. A mate-pair library that was prepared with 6 kb fragments yielded 6,492,803 reads. Approximately 5.8 Gb were qualified using the NGS QC Toolkit^29^ and constructed into contigs using Velvet^30^ and scaffolds using SSPACE^31^. Gaps were modified and closed using GapFiller^32^.

Repeat sequences in the assembled contigs were marked and removed using RepeatModeler and RepeatMasker^33^. Expressed sequence tags and protein sequences from phylogenetically related microorganisms were compared to *C. glabrata* CCTCC M202019 contigs using Exonerate^34^. Sequence matches were used for gene prediction with AUGUSTUS^35^. Functions of putative genes were annotated using the Kyoto Encyclopedia of Genes and Genomes Automatic Annotation Server (KOG)^36^,^37^ and Gene Ontology annotation^38^ tools. In addition, tRNA were identified using tRNAscan-SE^39^ and rRNA genes using RNAmmer^40^.

### Comparative genomics

A comparative genomic analysis between *C. glabrata* CCTCC M202019 and CBS 138 was performed by homology comparison of genes and detection of single nucleotide polymorphisms. Genes in the two strains were compared with the local nucleotide Basic Local Alignment Search Tool (BLASTN)^41^, and the alignment results were approximately divided into four categories, i.e., exactly the same, high homology with minimum standards of 90% identity and 80% length coverage, non-homologous relations with less than 90% identity or less than 80% length coverage, and completely non-matched genes.

Raw reads that were generated by the two pair-end libraries were filtered by the NGS QC Toolkit(available at http://www.nipgr.res.in/ngsqctoolkit.html), and then mapped to the 13 chromosomes of *C. glabrata* CBS138 using Bowtie 2^42^. The mapping files in the sequence alignment/map (SAM) format were converted, and the sorted and indexed binary versions of SAM files were generated using SAMtools^43^. Polymerase chain reaction duplicates that were generated during library construction were removed by Picard tools (http://picard.sourceforge.net). Since mapping errors occur frequently around known indels, the RealignerTargetCreator and IndelRealigner programs in the Genome Analysis Toolkit (GATK)^44^ software were used to reduce false-positives during SNP prediction. Finally, variant calling was recalibrated from the consistent results of the GATK and SAMtools. The SNPs in the Visual Component Framework format were annotated as a tab delimited file using the SNP Data Analysis Tool^45^, and they were located in specific genes using Artemis^46^ tools.

### Fermentation experiments

Four media were used to detect pyruvate production: SC medium (per liter, 100 g glucose and 6.7 g yeast nitrogen base without amino acids), YPD medium (per liter, 100 g glucose, 20 g peptone, and 10 g yeast extracts), BF medium^16^, and OF medium^47^. After sterilization at 115 °C for 10 min, 40 g·L^−1^ CaCO_3_ was added to buffer the pH.

*C. glabrata* CCTCC M202019 and CBS138 from agar slants^48^ were cultivated respectively in 50 mL and 500 mL of seed flask medium^48^ at 30 °C, with shaking at 200 rpm. Five milliliters of seed culture in the logarithmic growth phase was transferred into 50 mL of fermentation medium in 500 mL flasks. The fermentation broth was adjusted to an initial 5.5 pH, and the strains were cultivated for 48 h. The cell concentration^49^, and glucose^19^ and pyruvate^1^ contents were measured during the fermentation process.

### Virulence testing

#### Strains and cells

To compare the virulence of *C. glabrata* CCTCC M202019 and CBS138, the high virulent *C. albicans* SC5314 strain and the non-virulent *S. cerevisiae* BY4742 strain were chosen as positive and negative controls, respectively. Vascular endothelial cells were suspended by digestion with trypsin and counted using Trypan blue staining; greater than 95% of the cells were alive.

#### Media

Enzyme-producing medium contained (per liter) 20 g glucose, 10 g yeast extract, 20 g peptone, and 1 g KH_2_PO_4_, with an initial pH of 4.5. YGM2 milk medium contained (per liter) 20 g glucose, 10 g yeast extract, 1 g KH_2_PO_4_, and 15 g agar, with an initial pH of 4.5. After sterilization at 115 °C for 15 min, 1% (v/v) skimmed milk was added. Sabouraud’s dextrose medium contained (per liter) 40 g glucose and 10 g peptone.

#### Activation

Four experimental strains were activated using the enzyme-producing medium and then transferred to Columbia agar base. Both strains were cultivated at 37 °C for 24 h to recover their biological activities.

#### Protease activity

Single colonies of experimental strains that were cultivated on Columbia agar base and preserved at 4 °C were spotted onto YGM2 milk medium. After cultivation for 72 h at 37 °C, the diameter of the colonies and the surrounding transparent zones were measured. The protease activity (A) was equal to the diameter of the transparent zone divided by the colony diameter^50^.

#### Adherence assay

The concentrations of experimental strains and vascular endothelial cells were adjusted to 2 × 10^6^ mL^−1^ and 10^5^ mL^−1^, respectively, using Hanks’ balanced salt solution. The two were mixed in equal volumes (1 mL) and then incubated at 37 °C in 5% CO_2_ for 24 h. After staining and observation, the number of yeast adhering to more than three endothelial cells were counted, and the percentage of adherent cells calculated^51^.

Single colonies of the experimental strains were incubated in fresh Sabouraud’s dextrose medium at 37 °C, with shaking at 200 rpm. After reaching the late stage of growth, the number of cells was adjusted to 2 × 10^6^ mL^−1^, and 100 μL of a cell suspension was cultured for 4 h at 37 °C, with shaking 200 rpm. After discarding the medium and washing away non-adherent cells, 60 μL of 2,3-Bis-(2-methoxy-4-nitro-5-sulfophenyl)-2H-tetrazolium-5-carboxanilide was immediately added, and the 96-microwell plates were preserved in dark for 2 h. Adhesion capacity was determined by measuring the optical density at 490 nm^52^.

### *In silico* analysis of adhesin-like proteins in *C. glabrata* CCTCC M202019

The epithelial adhesin genes in *C. glabrata* CBS138 were collected from the *Candida* Genome Database (http://www.candidagenome.org/), and the *FLO* genes in *S. cerevisiae* mediating yeast flocculation and adherence to abiotic surfaces^25^,^53^ were used as the local BLASTN database. Additionally, proteins containing Pfam PF10528 domains in *C. glabrata* CCTCC M202019 were searched by the hmmscan command with an e-value less than 1e−05. The union genes were further submitted to the FungalRV^21^ and FaaPred^54^, and the two intersection was detected as adhesin proteins. These proteins’ domains were predicted with the SMART^55^ and PredGPI algorithms^56^. A phylogenetic tree of the adhesin-like proteins in *C. glabrata* CCTCC M202019 was constructed based on the clustering of the 1000 nucleotides from the 5′ end of EPA genes using BioEdit^57^.

## Conclusion

The pyruvate-producing strain *C. glabrata* CCTCC M202019 was sequenced and compared with the genome of *C. glabrata* CBS 138. Although the two strains generally had high similarity, genetic differences were identified mainly in central carbon metabolism and adhesion metabolism. Fermentation experiments and virulence testing supported the phenotypes of higher pyruvate producing and lower toxicity deduced from genotypes of *C. glabrata* CCTCC M202019. The study could be used for targeted improvements in pyruvate yield in *C. glabrata* and rationally engineering other industrial microorganism to produce pyruvate. Strategies for lowering the virulence of *C. glabrata* CBS 138 were suggested such as decreasing the number of low-complexity repeats of adhesins and mutating their functional domains. The data obtained in this study will facilitate the optimization of industrial bioprocess and further exploration of the pathogenicity of *C. glabrata*.

## Additional Information

**How to cite this article**: Xu, N. *et al*. Genome Sequencing of the Pyruvate-producing Strain *Candida glabrata* CCTCC M202019 and Genomic Comparison with Strain CBS138. *Sci. Rep.* **6**, 34893; doi: 10.1038/srep34893 (2016).

## Supplementary Material

Supplementary Information

Supplementary Dataset S1

## Figures and Tables

**Table 1:** Genome characteristics of *C. glabrata* CCTCC M202019.

General features	
Genome size (Mb)	12.1
GC content (%)	38.47
Number of contigs	111
Contig N50	659,495
Number of scaffolds	74
Scaffold N50	775,409
Length of classified repeats (%)	1.15
**Properties of gene annotation**	
Number of protein-coding sequences	5,345
Number of tRNA genes	191
Number of rRNA genes	6
Number of genes with EC assignment	961
Number of genes with GO assignment	4,788
Number of genes with KOG assignment	3,088

**Table 2:** Genetic differences related to pyruvate production in *C. glabrata.*

Subsystems	Function	Genes
Transport	glucose	HXT3 and HXT4/6/7
	niacin	TNR1 and TRN2
	acetic acid	CAGL0M03465g
	dicarboxylic acids	CAGL0J04114g
Oxidative phosphorylation	cytochrome C oxidase	COX1, COX2, COX7A, COX7C, and COX17
	cytochrome C reductase	CYTB and OCR10
	F-type ATPase	atp8, j, and k
	ETC complex IV	PET309
Downstream metabolism	acetyl-CoA hydrolase	ACH1 and GLN1
	glutamate synthase	GLT1
