# Supplementary material for: Genome Sequencing of the Pyruvate-producing Strain Candida glabrata CCTCC M202019 and Genomic Comparison with Strain CBS138
Source: Sci Rep. 2016 Oct 7;6:34893. doi: 10.1038/srep34893 (PMC5054605; doi:10.1038/srep34893)
Supplement: Supplementary Information [file srep34893-s1.pdf]

Genome Sequencing of the Pyruvate-producing Strain *Candida glabrata* CCTCC M202019 and Genomic Comparison with Strain CBS138

Nan Xu[1, 2], Chao Ye[1, 2], Xiulai Chen[1, 2], Jia Liu[1, 2], Liming Liu[1, 2]*, Jian Chen[1, 2]

[1]State Key Laboratory of Food Science and Technology, Jiangnan University, 1800 Lihu Road, Wuxi, Jiangsu 214122, China
[2]Key Laboratory of Industrial Biotechnology, Ministry of Education, School of Biotechnology, Jiangnan University, 1800 Lihu Road, Wuxi, Jiangsu 214122, China

*Correspondence should be addressed to Liming Liu (E-mail address: mingll@jiangnan.edu.cn)
Tel: +86-0510-85197875
Fax: +86-0510-85197875
Postal address: State Key Laboratory of Food Science and Technology, Jiangnan University, 1800 Lihu Road, Wuxi 214122

1. Evaluation of pyruvate production Capacity of *C. glabrata*

Supplementary Table S1 Comparison of Pyruvate Fermentation of *C. glabrata* CCTCC M202019 and CBS138

a: Fermentation parameters on synthetic complete (SC) medium

Parameters	Strains		Change (%) (A/B-1) x 100
	M202019	CBS138	
culture time (h)	60	60	0
dry cell weight ($g \cdot L^{-1}$)	6.11	7.52	-19
glucose consumption ($g \cdot L^{-1}$)	90.65	93.41	3
glucose consumption rate ($g \cdot L^{-1} \cdot h^{-1}$)	1.51	1.56	3
pyruvate production ($g \cdot L^{-1}$)	3.10	2.45	27
pyruvate productivity ($g \cdot L^{-1} \cdot h^{-1}$)	0.052	0.041	27
yield of pyruvate on glucose ($g \cdot g^{-1}$)	0.034	0.026	31

b: Fermentation parameters on yeast extract peptone dextrose (YPD) medium

Parameters	Strains		Change (%) (B/A-1) x 100
	M202019	CBS138	
culture time (h)	40	40	0
dry cell weight ($g \cdot L^{-1}$)	9.38	10.51	-11
glucose consumption ($g \cdot L^{-1}$)	100	100	0
glucose consumption rate ($g \cdot L^{-1} \cdot h^{-1}$)	2.5	2.5	0
pyruvate production ($g \cdot L^{-1}$)	2.31	1.71	35
pyruvate productivity ($g \cdot L^{-1} \cdot h^{-1}$)	0.058	0.043	35
yield of pyruvate on glucose ($g \cdot g^{-1}$)	0.23	0.17	35

c: Fermentation parameters on basal fermentation (BF) medium

Parameters	Strains		Change (%) (B/A-1) x 100
	M202019	CBS138	
culture time (h)	60	60	0
dry cell weight ($g \cdot L^{-1}$)	6.06	9.11	-33
glucose consumption ($g \cdot L^{-1}$)	100	100	0
glucose consumption rate ($g \cdot L^{-1} \cdot h^{-1}$)	1.67	1.67	0
pyruvate production ($g \cdot L^{-1}$)	35.76	29.45	21
pyruvate productivity ($g \cdot L^{-1} \cdot h^{-1}$)	0.60	0.49	21

yield of pyruvate on glucose ($g \cdot g^{-1}$)	3.58	2.95	21

d: Fermentation parameters on basal fermentation (BF) medium

Parameters	Strains		
	M202019	CBS138	Change (%) (B/A-1) x 100
culture time (h)	48	48	0
dry cell weight ($g \cdot L^{-1}$)	8.1	3.4	138
glucose consumption ($g \cdot L^{-1}$)	100	83.8	19
glucose consumption rate ($g \cdot L^{-1} \cdot h^{-1}$)	2.08	1.75	19
pyruvate production ($g \cdot L^{-1}$)	42.32	18.70	126
pyruvate productivity ($g \cdot L^{-1} \cdot h^{-1}$)	0.88	0.40	120
yield of pyruvate on glucose ($g \cdot g^{-1}$)	0.42	0.22	91

2. Quality control for the high throughput sequence data

a

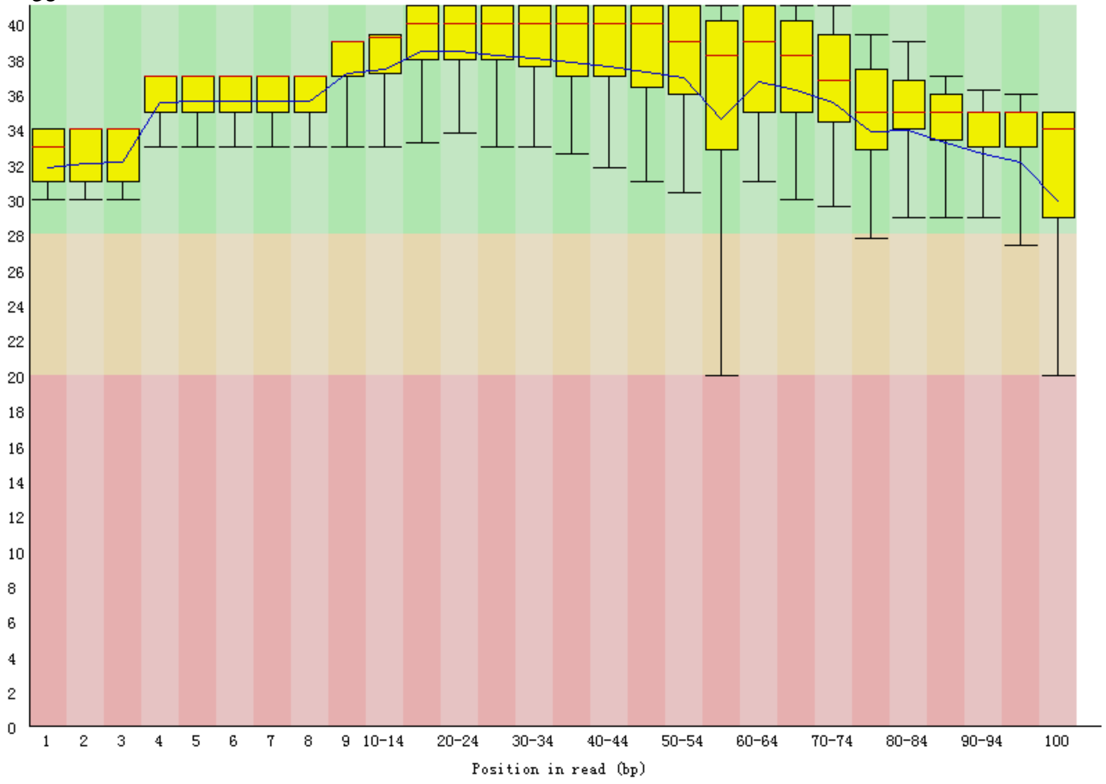

b

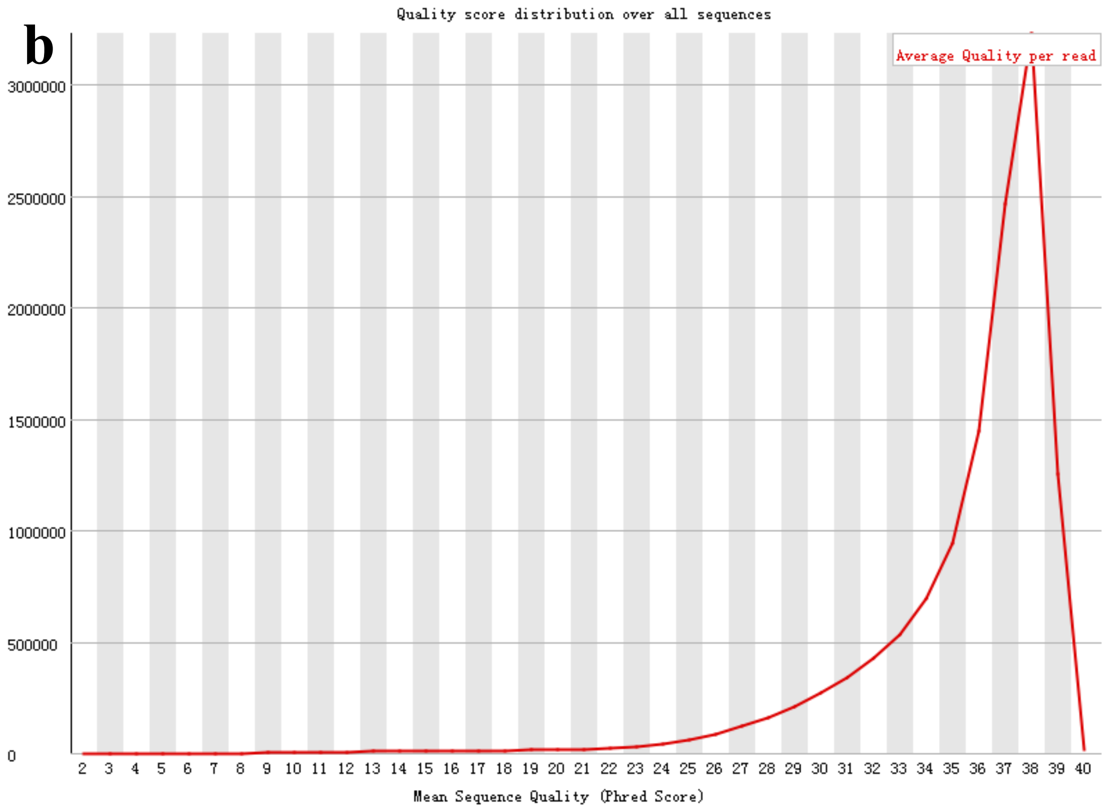
Quality score distribution over all sequences
Average Quality per read
3000000
2500000
2000000
1500000
1000000
500000
0
2 3 4 5 6 7 8 9 10 11 12 13 14 15 16 17 18 19 20 21 22 23 24 25 26 27 28 29 30 31 32 33 34 35 36 37 38 39 40
Mean Sequence Quality (Phred Score)

c

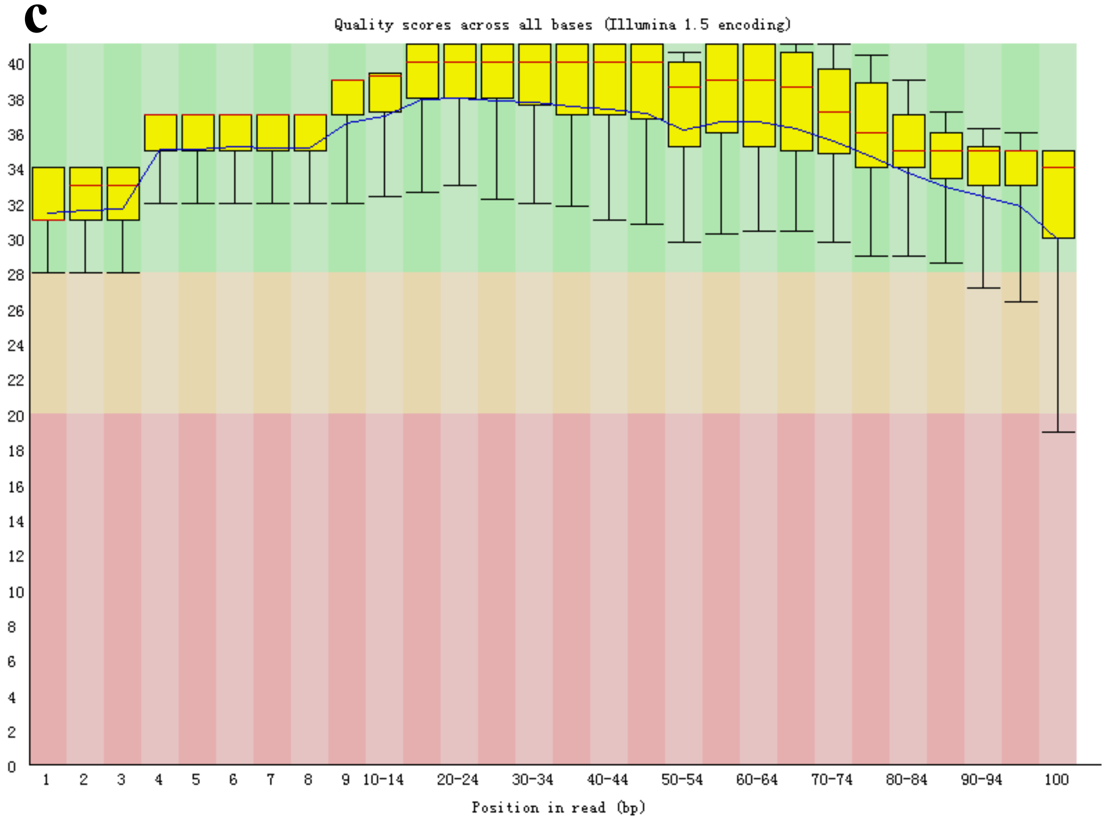
Quality scores across all bases (Illumina 1.5 encoding)
40
38
36
34
32
30
28
26
24
22
20
18
16
14
12
10
8
6
4
2
0
1 2 3 4 5 6 7 8 9 10-14 20-24 30-34 40-44 50-54 60-64 70-74 80-84 90-94 100
Position in read (bp)

d

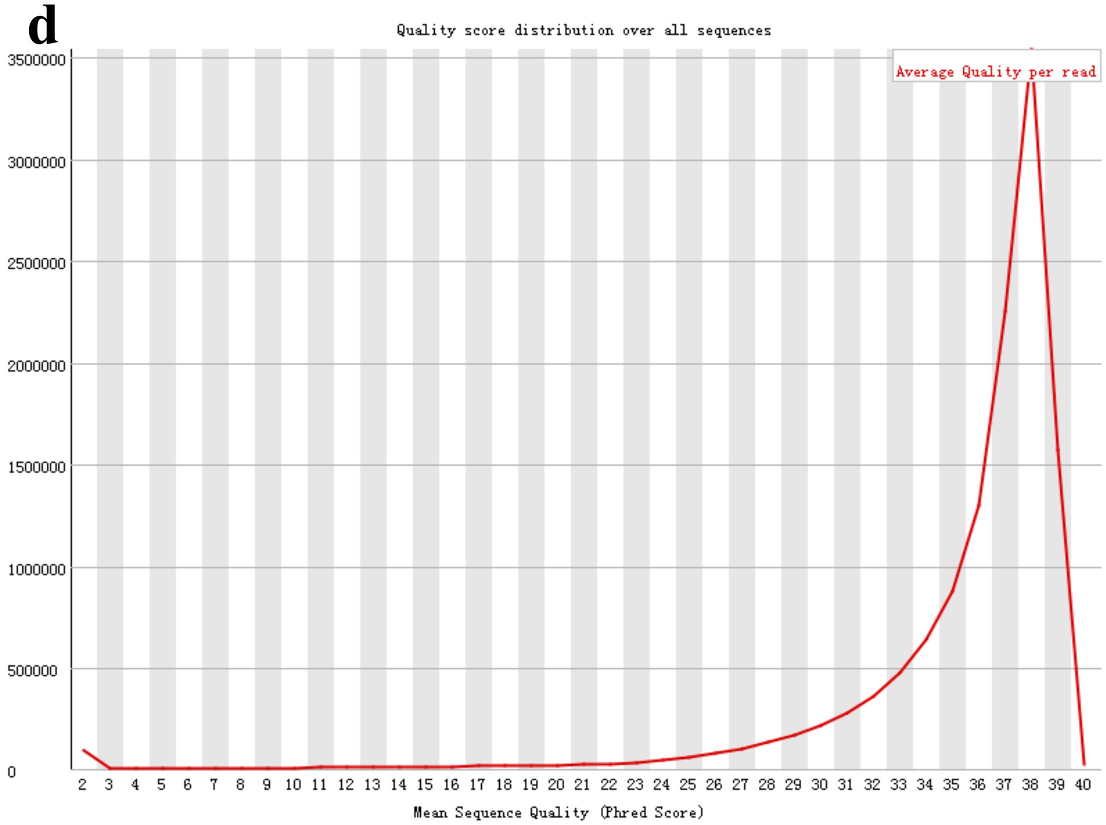
Quality score distribution over all sequences
Average Quality per read
3500000
3000000
2500000
2000000
1500000
1000000
500000
0
2 3 4 5 6 7 8 9 10 11 12 13 14 15 16 17 18 19 20 21 22 23 24 25 26 27 28 29 30 31 32 33 34 35 36 37 38 39 40
Mean Sequence Quality (Phred Score)

e

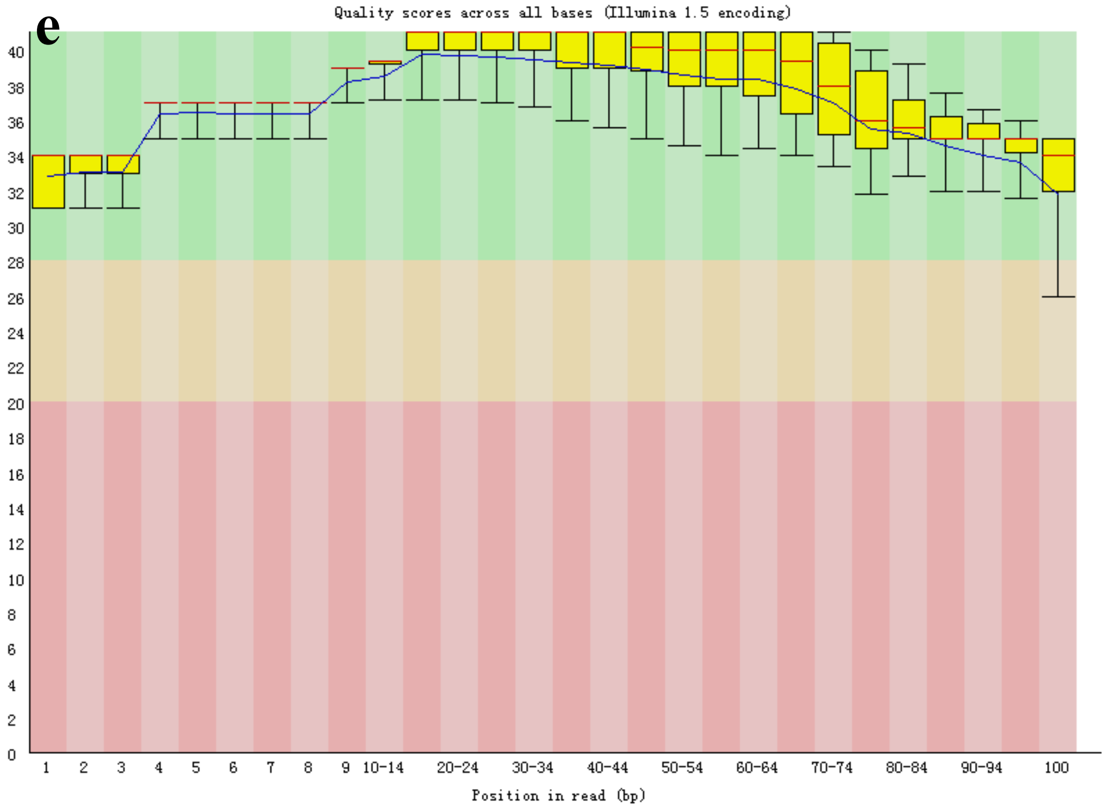
Quality scores across all bases (Illumina 1.5 encoding)
40
38
36
34
32
30
28
26
24
22
20
18
16
14
12
10
8
6
4
2
0
1 2 3 4 5 6 7 8 9 10-14 20-24 30-34 40-44 50-54 60-64 70-74 80-84 90-94 100
Position in read (bp)

f

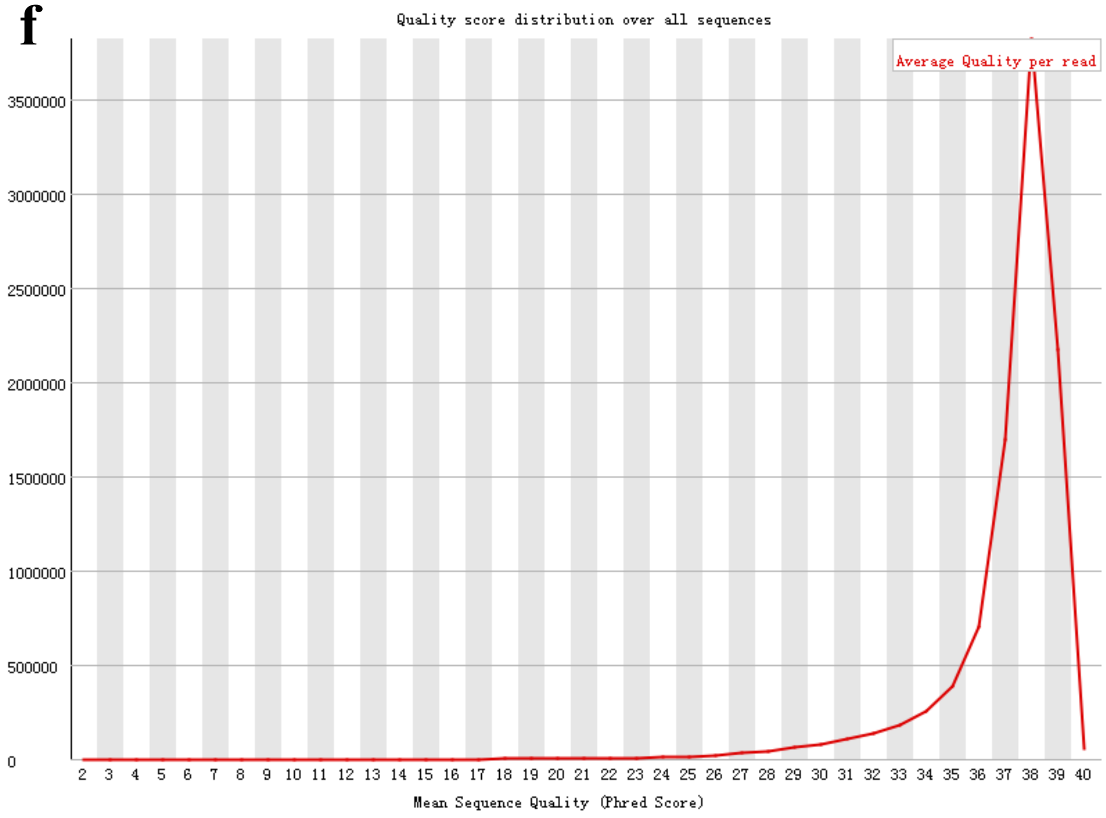
Quality score distribution over all sequences
Average Quality per read
3500000
3000000
2500000
2000000
1500000
1000000
500000
0
2 3 4 5 6 7 8 9 10 11 12 13 14 15 16 17 18 19 20 21 22 23 24 25 26 27 28 29 30 31 32 33 34 35 36 37 38 39 40
Mean Sequence Quality (Phred Score)

g

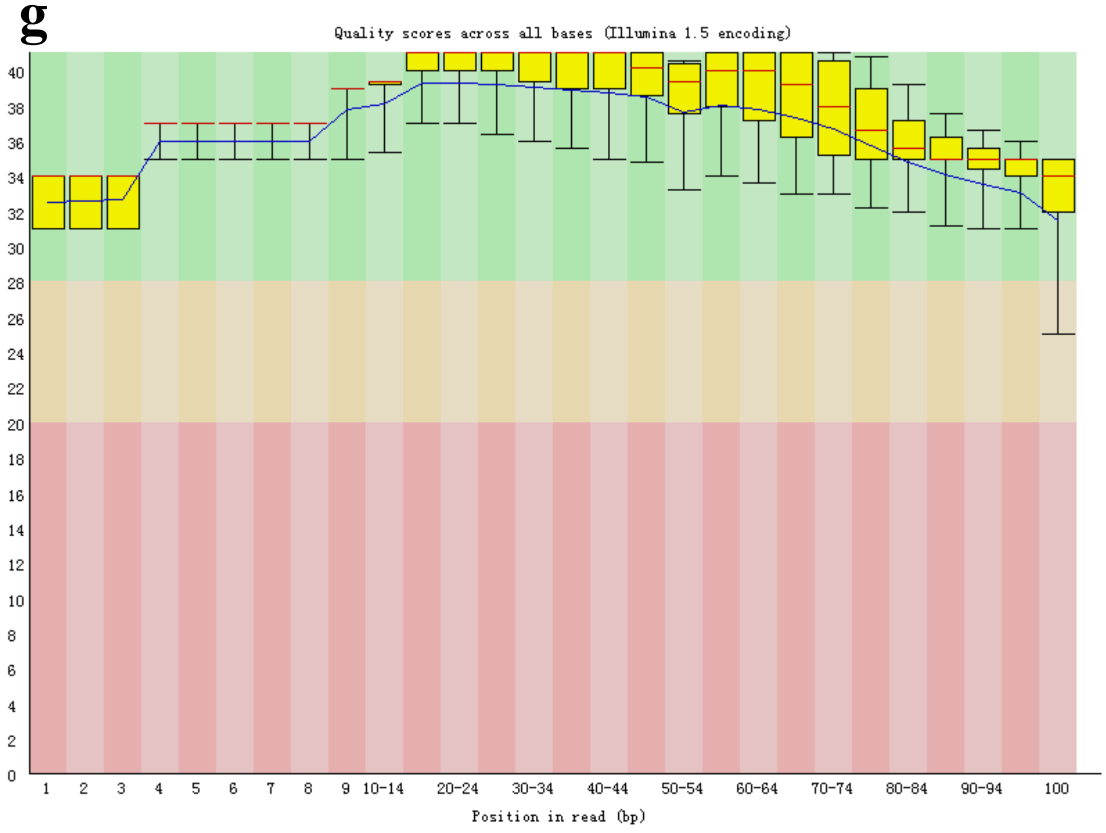
Quality scores across all bases (Illumina 1.5 encoding)
40 38 36 34 32 30 28 26 24 22 20 18 16 14 12 10 8 6 4 2 0
1 2 3 4 5 6 7 8 9 10-14 20-24 30-34 40-44 50-54 60-64 70-74 80-84 90-94 100
Position in read (bp)

h

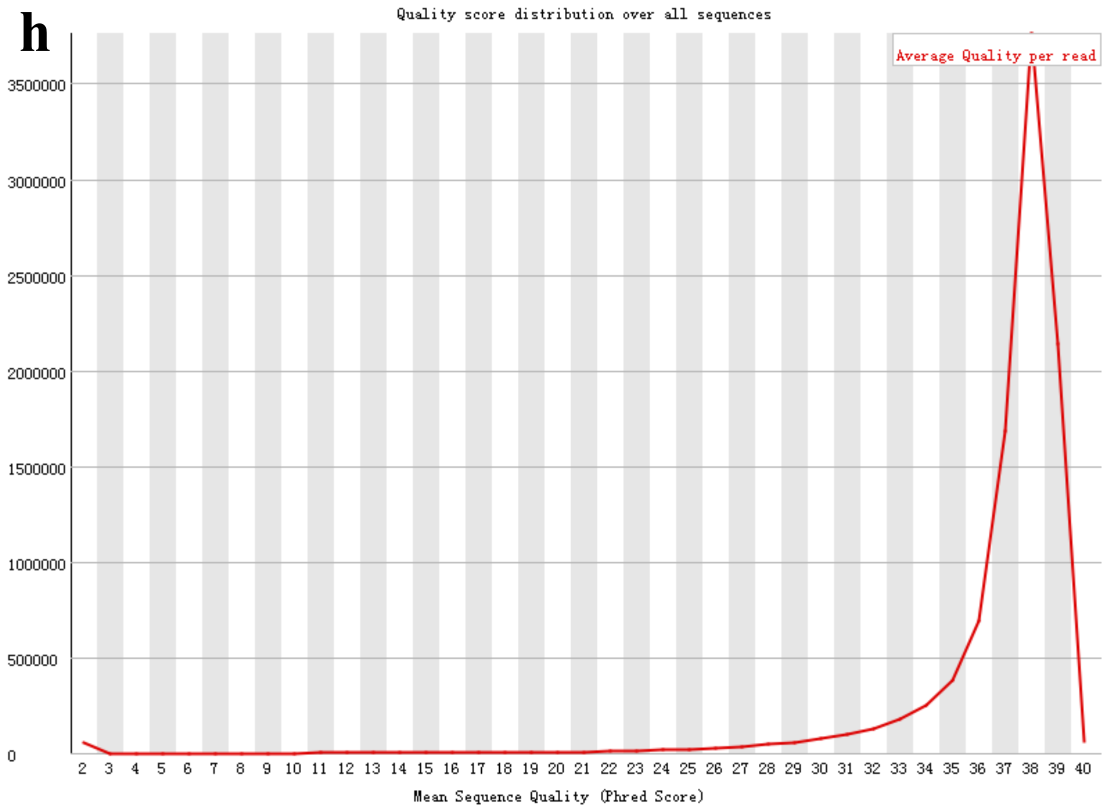

Quality score distribution over all sequences
Average Quality per read
3500000
3000000
2500000
2000000
1500000
1000000
500000
0
2 3 4 5 6 7 8 9 10 11 12 13 14 15 16 17 18 19 20 21 22 23 24 25 26 27 28 29 30 31 32 33 34 35 36 37 38 39 40
Mean Sequence Quality (Phred Score)

i

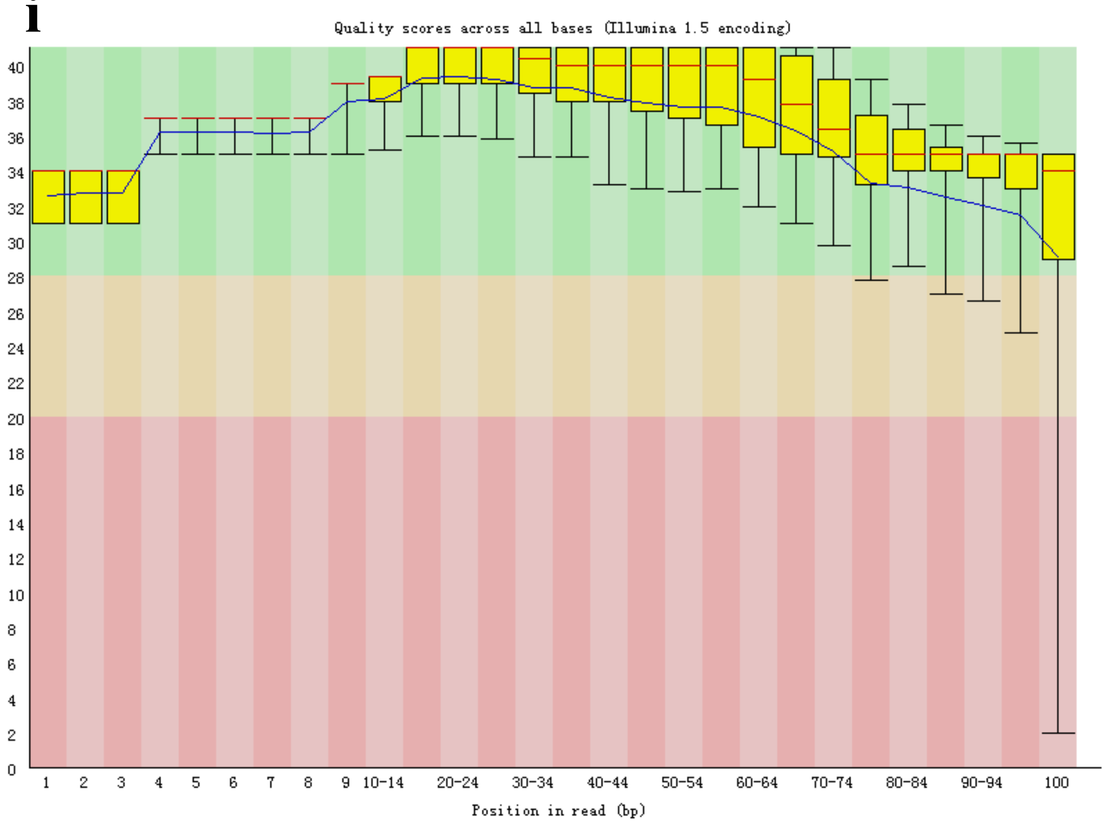

Quality scores across all bases (Illumina 1.5 encoding)
40 38 36 34 32 30 28 26 24 22 20 18 16 14 12 10 8 6 4 2 0
1 2 3 4 5 6 7 8 9 10-14 20-24 30-34 40-44 50-54 60-64 70-74 80-84 90-94 100
Position in read (bp)

j

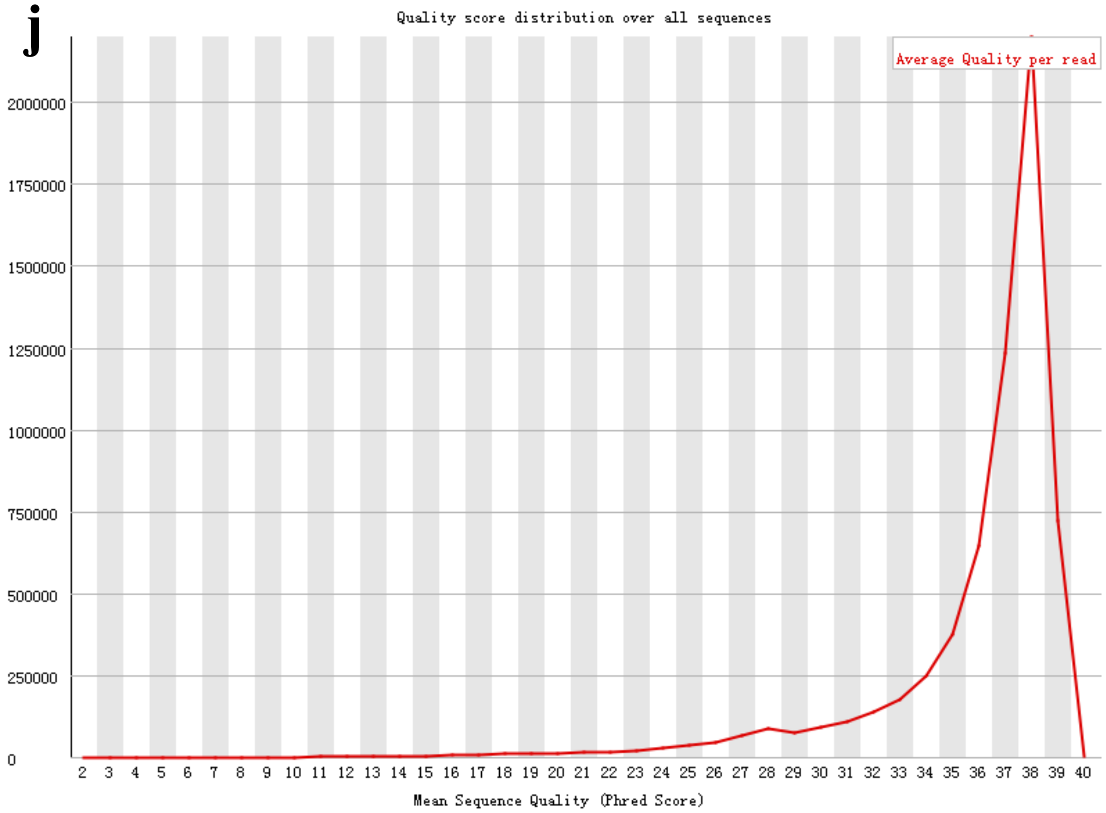
Quality score distribution over all sequences
Average Quality per read
2000000
1750000
1500000
1250000
1000000
750000
500000
250000
0
2 3 4 5 6 7 8 9 10 11 12 13 14 15 16 17 18 19 20 21 22 23 24 25 26 27 28 29 30 31 32 33 34 35 36 37 38 39 40
Mean Sequence Quality (Phred Score)

k

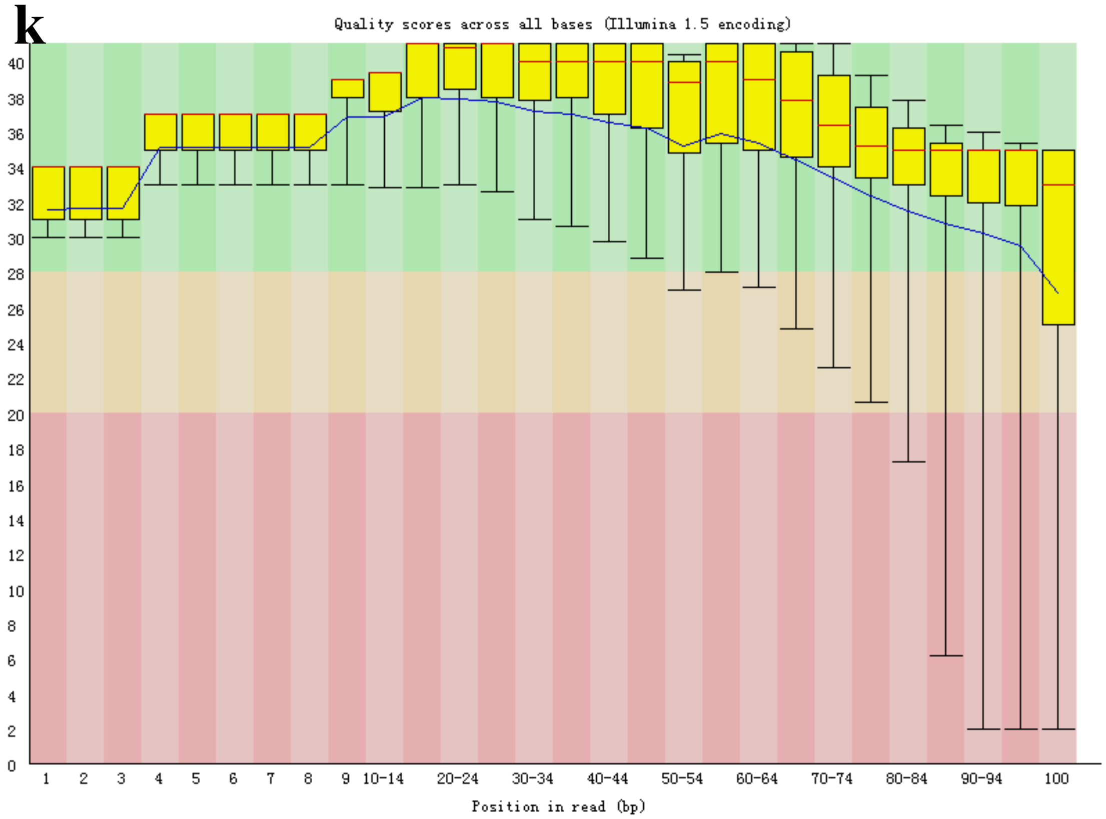
Quality scores across all bases (Illumina 1.5 encoding)
40
38
36
34
32
30
28
26
24
22
20
18
16
14
12
10
8
6
4
2
0
1 2 3 4 5 6 7 8 9 10-14 20-24 30-34 40-44 50-54 60-64 70-74 80-84 90-94 100
Position in read (bp)

l

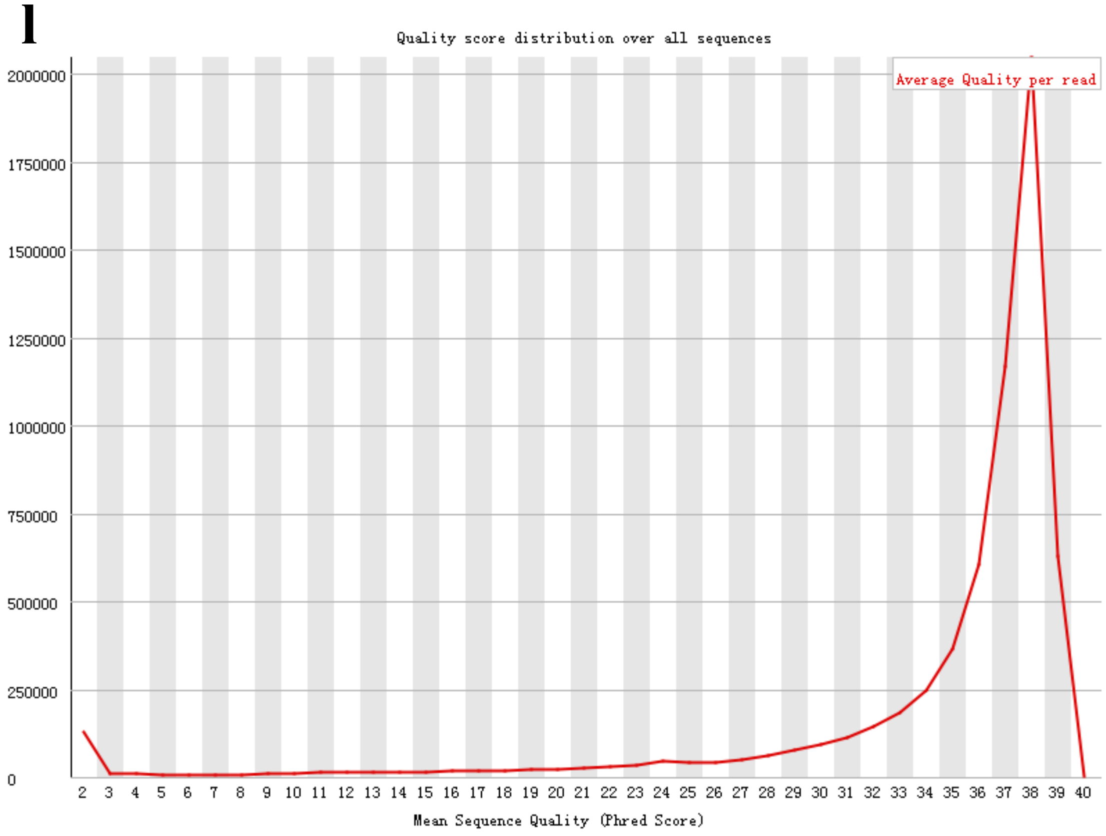

Supplementary Fig. S1 Per base and sequence quality distribution

All the NGS reads were used to calculate per base and sequence quality distribution. (a)-(d) for 150 bp pair-end library, (e)-(h) for 300 bp pair-end library, (i)-(l) for 6 kb mate-pair library. Except for the first base, lower quartile on the boxplot of per base was larger than 30. In addition, shown are the distributions in sequence quality, more than 90% reads got a higher than 35 score. Therefore, the *Candida glabrata* CCTCC M202019 genome was evaluated a high quality sequencing by FastQC [].

3. Genome annotation of *C. glabrata* CCTCC M202019 and comparative comparative genomics with *C. glabrata* CBS138

Supplementary Table S2 Distribution of repeat sequences in *C. glabrata* CCTCC M202019

Types	number of elements	length occupied	percentage of sequence
INEs:	16	1673bp	0.01%
ALUs	0	0bp	0%
MIRs	0	0bp	0%
LINEs:	0	0bp	0%

LINE1	0	0bp	0%
LINE2	0	0bp	0%
L3/CR1	0	0bp	0%
LTR elements:	0	0bp	0%
ERVL	0	0bp	0%
ERVL-MaLRs	0	0bp	0%
ERV_classI	0	0bp	0%
ERV_classII	0	0bp	0%
DNA elements:	0	0bp	0%
hAT-Charlie	0	0bp	0%
TcMar-Tigger	0	0bp	0%
Unclassified:	11	11449bp	0.09%
Total interspersed repeats:		13122bp	0.10%
Small RNA:	0	0bp	0%
Satellites:	0	0bp	0%
Simple repeats:	2644	121642bp	0.92%
Low complexity:	365	17220bp	0.13%

Gene function of *C. glabrata* CCTCC M202019 characterized by Gene Ontology, EuKaryotic Orthologous Groups, and Enzyme Classification number, tRNA and rRNA sequences, and common genetic traits of *C. glabrata* CCTCC M202019 and CBS138 were all list in ***Supplementary Dataset S1***.

Supplementary Table S3 Unique GO function classification in *C. glabrata* CCTCC M202019 and CBS138

	C. glabrata CCTCC M202019	*C. glabrata* CBS138
**B**	blastocyst development;	positive regulation of translational termination;
**i**	nucleosome disassembly;	positive regulation of cytoplasmic translational
**o**	positive regulation of asexual sporulation resulting in	elongation through polyproline stretches;
**l**	formation of a cellular spore;	DNA damage induced protein phosphorylation;
**o**	cellular carbohydrate catabolic process;	signal transduction in response to DNA damage;
**g**	nuclear-transcribed mRNA catabolic process, non-stop	ubiquitin homeostasis;
**i**	decay;	phagocytosis;
**c**	somatic stem cell population maintenance;	male meiosis I;
**a**	male germ-line stem cell population maintenance;	larval midgut cell programmed cell death;
**l**	protein localization to chromatin;	spermatid differentiation;
	cellular response to insulin stimulus;	termination of RNA polymerase III
**P**	maintenance of transcriptional fidelity during	transcription;

rocess	DNA-templated transcription elongation from RNA polymerase III promoter;<br>DNA geometric change;<br>cobalt ion transport;<br>cellular cobalt ion homeostasis;<br>[2Fe-2S] cluster assembly;<br>protein maturation by iron-sulfur cluster transfer;<br>positive regulation of mitochondrial DNA replication;	inositol phosphate dephosphorylation;<br>meiosis II cytokinesis;<br>embryo development ending in birth or egg hatching;<br>locomotion;
**cellular component**	RSC complex;<br>striated muscle thin filament;<br>nuclear euchromatin;<br>polytene chromosome;<br>polytene chromosome chromocenter;<br>site of double-strand break;	nuclear telomeric heterochromatin<br>nuclear rDNA heterochromatin<br>U1 snRNP<br>U2-type prespliceosome<br>mitochondrial proton-transporting ATP synthase complex, coupling factor F(o)
**molecular function**	L-lactate dehydrogenase (cytochrome) activity;<br>1-phosphatidylinositol 4-kinase activity;<br>inorganic cation transmembrane transporter activity;<br>iron-sulfur transferase activity;<br>epoxide hydrolase activity;	protease binding;<br>inositol monophosphate 1-phosphatase activity;<br>phospholipid binding;<br>uridylate kinase activity;<br>chitin synthase activity ;

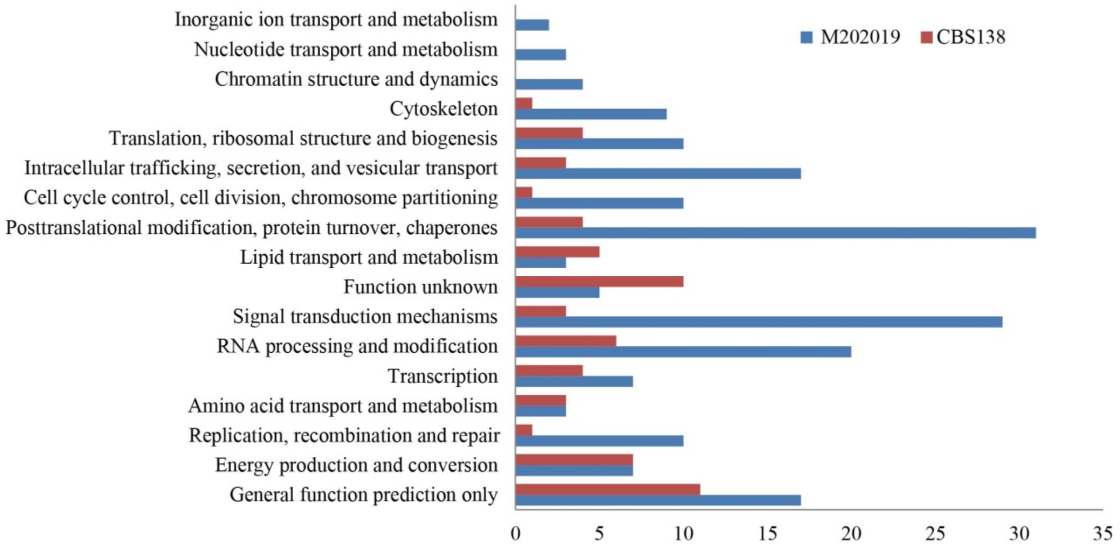

Supplementary Fig. S2 Unique KOG in *C. glabrata* CCTCC M202019 and CBS138

Supplementary Table S4 Genetic difference between in *C. glabrata* CCTCC M202019 and CBS138

C. glabrata CCTCC M202019			*C. glabrata* CBS138		
larger than 90% identity*	lower than 90% identity	unique genes	larger than 90% identity	lower than 90% identity	unique genes
CGRM202019g653.t1	CGRM202019g655.t1	CGRM202019g1982.t1	CAGL0C01837g	CAGL0A02211g	CAGL0B00264g
CGRM202019g665.t1	CGRM202019g2780.t1		CAGL0C01859g	CAGL0A02233g	CAGL0B01265g
CGRM202019g599.t1	CGRM202019g832.t1		CAGL0C04763g	CAGL0C00110g	CAGL0H05511g
CGRM202019g5035.t1	CGRM202019g3097.t1		CAGL0F02513g	CAGL0C00253g	CAGL0I01430g
CGRM202019g2547.t1	CGRM202019g836.t1		CAGL0F03091g	CAGL0C00847g	CAGL0M06501g
CGRM202019g2727.t1	CGRM202019g3967.t1		CAGL0F04961g	CAGL0C00968g	CAGL0C04411g
CGRM202019g2548.t1	CGRM202019g4868.t1		CAGL0G09713g	CAGL0C01133g	CAGL0D05082g
CGRM202019g1445.t1	CGRM202019g5036.t1		CAGL0C05379g	CAGL0C05379g	CAGL0C05379g
CGRM202019g4694.t1	CGRM202019g39.t1		CAGL0H06732g	CAGL0E01661g	CAGL0E06688g
CGRM202019g4744.t1	CGRM202019g1439.t1		CAGL0I04125g	CAGL0E05192g	CAGL0G10175g
CGRM202019g4498.t1	CGRM202019g1484.t1		CAGL0H05643g	CAGL0G10219g	CAGL0H05643g
CGRM202019g3868.t1	CGRM202019g1485.t1		CAGL0K02893g	CAGL0H00110g	CAGL0H08844g
CGRM202019g3306.t1	CGRM202019g2293.t2		CAGL0K07502g	CAGL0H10626g	
CGRM202019g2399.t1	CGRM202019g4182.t1		CAGL0L02255g	CAGL0I00220g	
CGRM202019g706.t1	CGRM202019g4658.t1		CAGL0L08110g	CAGL0I06182g	
CGRM202019g2171.t1	CGRM202019g1653.t1		CAGL0M10263g	CAGL0I07293g	
CGRM202019g2147.t1			CAGL0A00495g	CAGL0I10098g	
CGRM202019g2133.t1			CAGL0A01284g	CAGL0I10147g	
CGRM202019g2045.t1			CAGL0A01325g	CAGL0I10200g	
CGRM202019g378.t1			CAGL0A01366g	CAGL0I10246g	
CGRM202019g422.t1			CAGL0A01408g	CAGL0I10340g	
CGRM202019g432.t1			CAGL0A01474g	CAGL0I10362g	
CGRM202019g466.t1			CAGL0A01540g	CAGL0J01727g	
CGRM202019g479.t1			CAGL0A01782g	CAGL0J01774g	
CGRM202019g520.t1			CAGL0A01826g	CAGL0J02508g	
CGRM202019g524.t1			CAGL0A02145g	CAGL0J02552g	
CGRM202019g3165.t1			CAGL0A02321g	CAGL0J05159g	
CGRM202019g2903.t1			CAGL0A02651g	CAGL0J11891g	
CGRM202019g2851.t1			CAGL0A02794g	CAGL0J11968g	
CGRM202019g2743.t1			CAGL0A03278g	CAGL0K00170g	
CGRM202019g2755.t1			CAGL0A03872g	CAGL0K07546g	
CGRM202019g1056.t1			CAGL0A03993g	CAGL0K11440g	
CGRM202019g1007.t1			CAGL0B01203g	CAGL0K13024g	
CGRM202019g942.t1			CAGL0B01232g	CAGL0L00157g	
CGRM202019g941.t1			CAGL0B01837g	CAGL0L00227g	
CGRM202019g1193.t1			CAGL0B03399g	CAGL0L09911g	
CGRM202019g1189.t1			CAGL0C00209g	CAGL0L10092g	
CGRM202019g4466.t1			CAGL0C00539g	CAGL0L13299g	
CGRM202019g4359.t1			CAGL0C00781g	CAGL0L13332g	
CGRM202019g851.t1			CAGL0C01265g	CAGL0M00132g	
CGRM202019g1949.t1			CAGL0C02343g	CAGL0M08492g	

CGRM202019g1128.t1	CAGL0C03894g	CAGL0M12551g
CGRM202019g2402.t1	CAGL0C03938g	CAGL0M14069g
CGRM202019g2452.t1	CAGL0C03960g	
CGRM202019g2532.t1	CAGL0C05005g	
CGRM202019g3085.t1	CAGL0C05467g	
CGRM202019g3043.t1	CAGL0D01188g	
CGRM202019g749.t1	CAGL0D02090g	
CGRM202019g756.t1	CAGL0D03168g	
CGRM202019g774.t2	CAGL0D03234g	
CGRM202019g779.t2	CAGL0D04136g	
CGRM202019g2833.t1	CAGL0D06226g	
CGRM202019g2844.t1	CAGL0E00187g	
CGRM202019g2853.t2	CAGL0E01551g	
CGRM202019g2869.t2	CAGL0E02013g	
CGRM202019g2873.t2	CAGL0E04994g	
CGRM202019g2880.t2	CAGL0E05214g	
CGRM202019g2892.t1	CAGL0E05236g	
CGRM202019g2915.t1	CAGL0E06644g	
CGRM202019g2921.t1	CAGL0F00803g	
CGRM202019g2930.t1	CAGL0F01683g	
CGRM202019g2976.t1	CAGL0F03597g	
CGRM202019g833.t1	CAGL0F04609g	
CGRM202019g3021.t1	CAGL0F06457g	
CGRM202019g3050.t1	CAGL0F07249g	
CGRM202019g3070.t2	CAGL0F07381g	
CGRM202019g3074.t1	CAGL0F07579g	
CGRM202019g3079.t1	CAGL0F08987g	
CGRM202019g3117.t1	CAGL0G01276g	
CGRM202019g3118.t1	CAGL0G01452g	
CGRM202019g3234.t1	CAGL0G01496g	
CGRM202019g3239.t1	CAGL0G02475g	
CGRM202019g3305.t1	CAGL0G04125g	
CGRM202019g3319.t1	CAGL0G05830g	
CGRM202019g3393.t1	CAGL0G05940g	
CGRM202019g3397.t1	CAGL0G05984g	
CGRM202019g3415.t1	CAGL0G06842g	
CGRM202019g3442.t1	CAGL0G07183g	
CGRM202019g3480.t1	CAGL0G08646g	
CGRM202019g3481.t1	CAGL0G09130g	
CGRM202019g839.t1	CAGL0G09691g	
CGRM202019g853.t1	CAGL0G09757g	
CGRM202019g864.t2	CAGL0H02057g	
CGRM202019g870.t1	CAGL0H02123g	
CGRM202019g3486.t1	CAGL0H02189g	

CGRM202019g3493.t1	CAGL0H02783g
CGRM202019g3500.t1	CAGL0H03817g
CGRM202019g3507.t1	CAGL0H06281g
CGRM202019g3515.t1	CAGL0H08734g
CGRM202019g3516.t1	CAGL0H08932g
CGRM202019g3523.t1	CAGL0H09592g
CGRM202019g3539.t2	CAGL0H10274g
CGRM202019g3552.t1	CAGL0I01496g
CGRM202019g3578.t1	CAGL0I02816g
CGRM202019g3584.t1	CAGL0I02838g
CGRM202019g3585.t2	CAGL0I03828g
CGRM202019g939.t1	CAGL0I04928g
CGRM202019g1003.t1	CAGL0I06204g
CGRM202019g1022.t1	CAGL0J00737g
CGRM202019g1038.t2	CAGL0J01067g
CGRM202019g1042.t1	CAGL0J01800g
CGRM202019g1042.t2	CAGL0J01980g
CGRM202019g1044.t1	CAGL0J02266g
CGRM202019g1060.t2	CAGL0J02354g
CGRM202019g1062.t1	CAGL0J02530g
CGRM202019g959.t2	CAGL0J02794g
CGRM202019g967.t1	CAGL0J03234g
CGRM202019g982.t1	CAGL0J03780g
CGRM202019g982.t3	CAGL0J04114g
CGRM202019g1064.t1	CAGL0J04246g
CGRM202019g1068.t1	CAGL0J05764g
CGRM202019g3639.t1	CAGL0J09548g
CGRM202019g3674.t1	CAGL0J09702g
CGRM202019g3678.t1	CAGL0J10274g
CGRM202019g3688.t1	CAGL0J10670g
CGRM202019g3690.t1	CAGL0J10736g
CGRM202019g3704.t1	CAGL0K00110g
CGRM202019g3704.t1	CAGL0K00671g
CGRM202019g3705.t1	CAGL0K01507g
CGRM202019g3827.t1	CAGL0K05357g
CGRM202019g3896.t2	CAGL0K05467g
CGRM202019g3914.t2	CAGL0K05709g
CGRM202019g3938.t2	CAGL0K06149g
CGRM202019g3973.t1	CAGL0K06435g
CGRM202019g1225.t1	CAGL0K07700g
CGRM202019g1232.t1	CAGL0K09482g
CGRM202019g1238.t1	CAGL0K10252g
CGRM202019g1255.t1	CAGL0K10780g
CGRM202019g1258.t2	CAGL0K12034g

CGRM202019g1264.t1	CAGL0K12408g
CGRM202019g1270.t1	CAGL0K12562g
CGRM202019g1272.t1	CAGL0K12694g
CGRM202019g1293.t1	CAGL0L00385g
CGRM202019g3990.t1	CAGL0L00407g
CGRM202019g4018.t1	CAGL0L01089g
CGRM202019g4042.t2	CAGL0L01639g
CGRM202019g4093.t2	CAGL0L02585g
CGRM202019g4096.t2	CAGL0L06050g
CGRM202019g4117.t2	CAGL0L06424g
CGRM202019g4189.t2	CAGL0L06644g
CGRM202019g4191.t1	CAGL0L06886g
CGRM202019g4202.t1	CAGL0L08624g
CGRM202019g4219.t2	CAGL0L11462g
CGRM202019g4221.t2	CAGL0L11484g
CGRM202019g4271.t1	CAGL0L11770g
CGRM202019g4322.t1	CAGL0L11814g
CGRM202019g4368.t1	CAGL0L12760g
CGRM202019g4376.t2	CAGL0L13354g
CGRM202019g4377.t1	CAGL0M02739g
CGRM202019g4385.t2	CAGL0M03465g
CGRM202019g4417.t1	CAGL0M03861g
CGRM202019g4418.t2	CAGL0M06523g
CGRM202019g4418.t3	CAGL0M09317g
CGRM202019g4456.t2	CAGL0M10890g
CGRM202019g4559.t1	CAGL0M14113g
CGRM202019g4604.t1	CAGL0C03828g
CGRM202019g1372.t1	CAGL0E00341g
CGRM202019g1411.t1	CAGL0J03245g
CGRM202019g4607.t1	CAGL0G07040g
CGRM202019g1435.t1	
CGRM202019g4608.t1	
CGRM202019g4654.t1	
CGRM202019g4654.t1	
CGRM202019g4655.t1	
CGRM202019g4656.t1	
CGRM202019g4657.t1	
CGRM202019g4670.t1	
CGRM202019g4670.t1	
CGRM202019g4682.t1	
CGRM202019g4718.t2	
CGRM202019g4740.t1	
CGRM202019g4741.t1	
CGRM202019g4777.t2	

CGRM202019g4790.t2
CGRM202019g4798.t2
CGRM202019g4808.t1
CGRM202019g4854.t2
CGRM202019g4859.t1
CGRM202019g4871.t1
CGRM202019g4872.t1
CGRM202019g4887.t2
CGRM202019g4897.t1
CGRM202019g4941.t1
CGRM202019g4952.t2
CGRM202019g4953.t1
CGRM202019g4962.t2
CGRM202019g4965.t1
CGRM202019g4965.t2
CGRM202019g4975.t1
CGRM202019g4991.t2
CGRM202019g5021.t2
CGRM202019g5032.t3
CGRM202019g5037.t1
CGRM202019g5038.t1
CGRM202019g51.t1
CGRM202019g67.t1
CGRM202019g197.t1
CGRM202019g1561.t1
CGRM202019g1564.t2
CGRM202019g1567.t1
CGRM202019g1641.t2
CGRM202019g1694.t1
CGRM202019g1697.t1
CGRM202019g1699.t2
CGRM202019g1726.t1
CGRM202019g1757.t2
CGRM202019g1797.t2
CGRM202019g1802.t1
CGRM202019g1855.t2
CGRM202019g1885.t2
CGRM202019g1889.t2
CGRM202019g1898.t2
CGRM202019g1917.t2
CGRM202019g1919.t1
CGRM202019g1923.t2
CGRM202019g1947.t2
CGRM202019g1951.t1

CGRM202019g1951.t2
CGRM202019g1978.t1
CGRM202019g1979.t1
CGRM202019g209.t1
CGRM202019g335.t1
CGRM202019g346.t1
CGRM202019g1995.t2
CGRM202019g2015.t2
CGRM202019g2041.t1
CGRM202019g2062.t2
CGRM202019g2071.t2
CGRM202019g2115.t2
CGRM202019g2118.t2
CGRM202019g2160.t2
CGRM202019g2167.t2
CGRM202019g2202.t1
CGRM202019g2219.t1
CGRM202019g2236.t1
CGRM202019g2242.t2
CGRM202019g2248.t1
CGRM202019g2248.t2
CGRM202019g2248.t3
CGRM202019g2261.t2
CGRM202019g2291.t2
CGRM202019g2300.t1
CGRM202019g2305.t1
CGRM202019g2312.t1
CGRM202019g2331.t1
CGRM202019g2358.t1
CGRM202019g2374.t1
CGRM202019g2380.t1
CGRM202019g2401.t1
CGRM202019g406.t2
CGRM202019g408.t1
CGRM202019g427.t1
CGRM202019g452.t1
CGRM202019g459.t1
CGRM202019g548.t2
CGRM202019g570.t2
CGRM202019g572.t2
CGRM202019g626.t1
CGRM202019g2410.t1
CGRM202019g2423.t1
CGRM202019g2462.t2

CGRM202019g2519.t2
CGRM202019g2571.t1
CGRM202019g2592.t1
CGRM202019g2606.t2
CGRM202019g2652.t1
CGRM202019g2693.t2
CGRM202019g2698.t1
CGRM202019g2724.t1
CGRM202019g807.t1
CGRM202019g1414.t1
CGRM202019g4869.t1
CGRM202019g1474.t1
CGRM202019g1525.t1

* the identity gotten from the local blast

Supplementary Table S5 The distribution of SNP in *C. glabrata* CBS138

Chromosome	Position	SNP	Within feature	Chromosome	Position	SNP	Within feature
ChrA	2743	GC -> G	CAGL0A00110g	ChrH	6294	G -> T	CAGL0H00132g
ChrA	56909	C -> T	CAGL0A00495g	ChrH	6311	G -> C	CAGL0H00132g
ChrA	170452	T -> C	CAGL0A01716g	ChrH	6323	G -> C	CAGL0H00132g
ChrA	236422	T -> C	CAGL0A02233g	ChrH	6335	G -> C	CAGL0H00132g
ChrA	236429	G -> A	CAGL0A02233g	ChrH	6719	CT -> C	CAGL0H00132g
ChrA	236438	G -> T	CAGL0A02233g	ChrH	6844	AC -> A	CAGL0H00132g
ChrA	236443	C -> G	CAGL0A02233g	ChrH	6902	TG -> T	CAGL0H00132g
ChrB	1857	T -> TG	CAGL0B00110g	ChrH	7020	C -> T	CAGL0H00132g
ChrB	2459	CT -> C	CAGL0B00110g	ChrH	7041	T -> A	CAGL0H00132g
ChrB	2657	AC -> A	CAGL0B00110g	ChrH	7071	AC -> A	CAGL0H00132g
ChrB	2673	GT -> G	CAGL0B00110g	ChrH	7118	TA -> T	CAGL0H00132g
ChrB	3151	G -> A	CAGL0B00110g	ChrH	7207	TG -> T	CAGL0H00132g
ChrB	3301	AT -> A	CAGL0B00110g	ChrH	246353	CT -> C	CAGL0H02695g
ChrB	3403	CT -> C	CAGL0B00110g	ChrH	358783	G -> A	CAGL0H03817g
ChrB	3572	CA -> C	CAGL0B00110g	ChrH	621158	T -> A	CAGL0H06281g

ChrB	3672	CT -> C	CAGL0B00110g	ChrH	963528	T -> C	CAGL0H09878g
ChrB	3687	GC -> G	CAGL0B00110g	ChrH	965488	A -> G	CAGL0H09900r
ChrB	3918	GA -> G	CAGL0B00110g	ChrI	6931	CG -> C	CAGL0I00110g
ChrB	4010	CG -> C	CAGL0B00110g	ChrI	6936	CA -> C	CAGL0I00110g
ChrB	331627	C -> T	CAGL0B03421g	ChrI	7073	GT -> G	CAGL0I00110g
ChrB	361566	A -> AG	CAGL0B03619g	ChrI	7092	GC -> G	CAGL0I00110g
ChrB	498888	TA -> T	CAGL0B05093g	ChrI	7121	TC -> T	CAGL0I00110g
ChrB	499013	AC -> A	CAGL0B05093g	ChrI	7129	TG -> T	CAGL0I00110g
ChrB	499124	AG -> A	CAGL0B05093g	ChrI	248677	TA -> T	CAGL0I02816g
ChrB	499197	G -> C	CAGL0B05093g	ChrI	248722	CG -> C	CAGL0I02838g
ChrB	499200	G -> A	CAGL0B05093g	ChrI	1023356	C -> A	CAGL0I10362g
ChrB	499201	G -> C	CAGL0B05093g	ChrJ	2861	CA -> C	CAGL0J00110g
ChrB	499267	T -> C	CAGL0B05093g	ChrJ	2925	AG -> A	CAGL0J00110g
ChrB	499276	CG -> C	CAGL0B05093g	ChrJ	2961	G -> A	CAGL0J00110g
ChrB	499871	AC -> A	CAGL0B05093g	ChrJ	2973	C -> T	CAGL0J00110g
ChrB	501343	AC -> A	CAGL0B05093g	ChrJ	2979	G -> C	CAGL0J00110g
ChrC	347	C -> T	CAGL0C00110g	ChrJ	104494	C -> G	CAGL0J01067g
ChrC	94576	A -> C	CAGL0C00968g	ChrJ	387161	AC -> A	CAGL0J04114g
ChrC	96027	T -> C	CAGL0C00968g	ChrJ	387164	CA -> C	CAGL0J04114g
ChrC	97243	G -> T	CAGL0C00968g	ChrJ	387196	AC -> A	CAGL0J04114g
ChrC	98119	G -> A	CAGL0C00968g	ChrJ	398441	C -> A	CAGL0J04268g
ChrC	107657	T -> G	CAGL0C01067g	ChrJ	613639	TGG -> T,TG	CAGL0J06402g
ChrC	107669	T -> G	CAGL0C01067g	ChrJ	953829	C -> CCG	CAGL0J09702g
ChrC	107800	AT -> A	CAGL0C01067g	ChrJ	1004938	C -> T	CAGL0J10274g
ChrC	107902	C -> T	CAGL0C01067g	ChrJ	1049031	G -> C	CAGL0J10736g

ChrC	112014	C -> G	CAGL0C01133g	ChrJ	1188865	CA -> C	CAGL0J11990g
ChrC	112016	A -> T	CAGL0C01133g	ChrJ	1189480	C -> T	CAGL0J11990g
ChrC	112019	T -> C	CAGL0C01133g	ChrJ	1189611	CG -> C	CAGL0J11990g
ChrC	112130	A -> C	CAGL0C01133g	ChrJ	1189634	GT -> G	CAGL0J11990g
ChrC	112157	T -> G	CAGL0C01133g	ChrJ	1191467	T -> C	CAGL0J11990g
ChrC	112160	G -> A	CAGL0C01133g	ChrJ	1194022	T -> C	CAGL0J11990g
ChrC	112163	C -> T	CAGL0C01133g	ChrK	95456	ATC -> A	CAGL0K00935g
ChrC	112704	A -> G	CAGL0C01133g	ChrK	132355	G -> T	CAGL0K01507g
ChrC	112751	A -> G	CAGL0C01133g	ChrK	316816	G -> A	CAGL0K03443g
ChrC	112862	C -> A	CAGL0C01133g	ChrK	526089	A -> G	CAGL0K05357g
ChrC	382148	A -> C	CAGL0C03894g	ChrK	532195	CA -> C	CAGL0K05423g
ChrD	3937	C -> G	CAGL0D00110g	ChrK	556803	C -> T	CAGL0K05709g
ChrD	3941	AC -> A	CAGL0D00110g	ChrK	742415	T -> C	CAGL0K07502g
ChrD	4462	GT -> G	CAGL0D00110g	ChrK	742444	A -> C	CAGL0K07502g
ChrD	4572	GT -> G	CAGL0D00110g	ChrK	742449	AT -> A	CAGL0K07502g
ChrD	4636	GT -> G	CAGL0D00110g	ChrK	742467	T -> C	CAGL0K07502g
ChrD	4641	T -> A	CAGL0D00110g	ChrK	742513	G -> C	CAGL0K07502g
ChrD	328228	A -> G	CAGL0D03168g	ChrK	742519	T -> C	CAGL0K07502g
ChrD	335260	T -> C	CAGL0D03234g	ChrK	742527	T -> C	CAGL0K07502g
ChrD	335844	C -> T	CAGL0D03234g	ChrK	742535	T -> C	CAGL0K07502g
ChrD	650725	G -> T	CAGL0D06732g	ChrK	742549	T -> C	CAGL0K07502g
ChrD	650733	G -> T	CAGL0D06732g	ChrK	742581	A -> C	CAGL0K07502g
ChrD	650759	CA -> C	CAGL0D06732g	ChrK	742583	T -> C	CAGL0K07502g
ChrD	650843	G -> C	CAGL0D06732g	ChrK	742602	A -> C	CAGL0K07502g
ChrD	650847	TA -> T	CAGL0D06732g	ChrK	744341	TG -> T	CAGL0K07524g

ChrE	2171	G -> T	CAGL0E00110g	ChrK	1000743	GAT -> G	CAGL0K10252g
ChrE	4513	C -> CG	CAGL0E00110g	ChrK	1130725	CG -> C	CAGL0K11748g
ChrE	4632	CG -> C	CAGL0E00110g	ChrK	1164388	G -> A	CAGL0K12034g
ChrE	10907	C -> T	CAGL0E00187g	ChrK	1212182	A -> G	CAGL0K12408g
ChrF	2306	G -> GT	CAGL0F00110g	ChrK	1236292	G -> T	CAGL0K12562g
ChrF	3307	TG -> T	CAGL0F00110g	ChrL	19512	A -> G	CAGL0L00227g
ChrF	3932	AC -> A	CAGL0F00110g	ChrL	44407	G -> T	CAGL0L00407g
ChrF	4408	TA -> T	CAGL0F00110g	ChrL	124391	G -> T	CAGL0L01089g
ChrF	90968	T -> G	CAGL0F00803g	ChrL	177404	C -> G	CAGL0L01595r
ChrF	586422	AG -> A	CAGL0F05819g	ChrL	178927	A -> AT	CAGL0L01639g
ChrF	605178	T -> C	CAGL0F06061g	ChrL	235154	GTT -> G,GT	CAGL0L01991g
ChrF	926389	TA -> T	CAGL0F09273g	ChrL	305419	A -> C	CAGL0L02585g
ChrF	926524	AT -> A	CAGL0F09273g	ChrL	417188	TC -> T	CAGL0L03630g
ChrF	926725	CT -> C	CAGL0F09273g	ChrL	587459	TC -> T	CAGL0L05236g
ChrG	4317	GA -> G	CAGL0G00110g	ChrL	604117	G -> T	CAGL0L05500g
ChrG	4360	AT -> A	CAGL0G00110g	ChrL	939562	A -> T	CAGL0L08624g
ChrG	142051	C -> G	CAGL0G01496g	ChrL	941345	A -> C	CAGL0L08624g
ChrG	268420	AC -> A	CAGL0G02915g	ChrL	1072858	A -> C	CAGL0L09955g
ChrG	653876	A -> C	CAGL0G06842g	ChrL	1263007	T -> C	CAGL0L11792g
ChrG	653922	A -> C	CAGL0G06842g	ChrL	1266574	A -> T	CAGL0L11792g
ChrG	653994	A -> C	CAGL0G06842g	ChrL	1455011	A -> G	CAGL0L13398r
ChrG	670054	C -> CA	CAGL0G07040g	ChrM	393322	AAT -> A	CAGL0M03465g
ChrG	760822	CA -> C	CAGL0G08041g	ChrM	613506	G -> T	CAGL0M05797g
ChrG	815281	T -> G	CAGL0G08646g	ChrM	659487	T -> TC	CAGL0M06325g
ChrG	931981	C -> A	CAGL0G09757g	ChrM	926262	A -> G	CAGL0M09317g

ChrH	4673	AG -> A	CAGL0H00132g	ChrM	1033820	A -> C	CAGL0M10329g
ChrH	6287	A -> T	CAGL0H00132g	ChrM	1245765	AG -> A	CAGL0M12496g
ChrH	6293	G -> C	CAGL0H00132g				

Supplementary Table S6 Genes with SNPs located in their CDS

Genes	Number of SNPs	Gene Function
CAGL0H00132g	15	Adhesin-like gene with 4 tandem repeats; possible pseudogene
CAGL0B00110g	12	Predicted adhesin-like protein
CAGL0K07502g	12	Similarity possibly noncoding
CAGL0B05093g	10	Protein with tandem repeats
CAGL0D00110g	6	Pseudogene with a predicted GPI-anchor; putative adhesin-like protein
CAGL0I00110g	6	Adhesin-like pseudogene with multiple tandem repeats; predicted GPI-anchor
CAGL0D06732g	5	EPA21,Pseudogene with a predicted GPI-anchor sequence; putative adhesin-like protein
CAGL0J00110g	5	Putative adhesin-like protein
CAGL0J11990g	5	Adhesin-like gene with internal repeats; predicted GPI-anchor; predicted pseudogene
CAGL0A02233g	4	Strain CBS138 chromosome A complete sequence
CAGL0C01067g	4	Predicted adhesin-like protein
CAGL0F00110g	4	Pseudogene with internal repeats; putative adhesin-like protein; predicted GPI-anchor
CAGL0E00110g	3	Agglutinin-like protein with tandem repeats; predicted GPI-anchor
CAGL0G06842g	3	Similar to uniprot|P47068 Saccharomyces cerevisiae YJL020c
CAGL0J04114g	3	Strain CBS138 chromosome J complete sequence
CAGL0C00968g	2	Similarities with uniprot|Q08294 Saccharomyces cerevisiae YOL155c
CAGL0D03234g	2	Strain CBS138 chromosome D complete sequence
CAGL0G00110g	2	Pseudogene with internal repeats; putative adhesin; predicted GPI-anchor
CAGL0L11792g	2	Polyadenylate-binding protein, cytoplasmic and nuclear (PABP) (Poly(A)-binding protein) (Polyadenylate tail-binding protein)
CAGL0A00110g	1	EPA19 putative adhesin; predicted GPI-anchor
CAGL0A00495g	1	Strain CBS138 chromosome A complete sequence
CAGL0B03421g	1	Strain CBS138 chromosome B complete sequence
CAGL0C03894g	1	Strain CBS138 chromosome C complete sequence
CAGL0H00132g	1	Similar to uniprot|P08678 Saccharomyces cerevisiae YJL005w CYR1 adenylate cyclase

CAGL0H03817g	1	Putative adhesin-like protein
CAGL0H06281g	1	Conserved oligomeric Golgi complex subunit 6 (COG complex subunit 6) (Component of oligomeric Golgi complex 6)
CAGL0H09900r	1	Strain CBS138 chromosome G complete sequence
CAGL0I00110g	1	Similar to uniprot|Q03195 Saccharomyces cerevisiae YDR091c
CAGL0I02816g	1	Transcriptional activator POG1
CAGL0I02838g	1	Similarities with uniprot|Q05854 Saccharomyces cerevisiae YLR278c
CAGL0I10362g	1	Similar to uniprot|P25374 Saccharomyces cerevisiae YCL017c NFS1
CAGL0J00110g	1	Similar to uniprot|P31383 Saccharomyces cerevisiae YAL016w TPD3
CAGL0J01067g	1	tRNA-Val
CAGL0J04114g	1	Strain CBS138 chromosome I complete sequence
CAGL0J04268g	1	Strain CBS138 chromosome I complete sequence
CAGL0J09702g	1	Similarity
CAGL0J10274g	1	Similar to uniprot|Q00618 Saccharomyces cerevisiae YJL031c BET4
CAGL0J10736g	1	Acetyl-CoA hydrolase (EC 3.1.2.1) (Acetyl-CoA deacylase) (Acetyl-CoA acylase)
CAGL0J11990g	1	Similar to uniprot|Q07622 Saccharomyces cerevisiae YDL203c
CAGL0K01507g	1	Strain CBS138 chromosome J complete sequence
CAGL0K05357g	1	Protein phosphatase methylesterase 1 (PME-1) (EC 3.1.1.89)
CAGL0K05709g	1	Strain CBS138 chromosome K complete sequence
CAGL0K07502g	1	Glutamine synthetase (GS) (EC 6.3.1.2) (Glutamate--ammonia ligase)
CAGL0K07524g	1	Similar to uniprot|Q12263 Saccharomyces cerevisiae YDR507c GIN4
CAGL0K10252g	1	Similar to uniprot|P36069 Saccharomyces cerevisiae YKL128c suppressor of TPS2 mutant
CAGL0K12034g	1	Ubiquitin carboxyl-terminal hydrolase (EC 3.4.19.12)
CAGL0K12408g	1	Strain CBS138 chromosome K complete sequence
CAGL0K12562g	1	GPI inositol-deacylase (EC 3.1.-.-)
CAGL0L00227g	1	Strain CBS138 chromosome K complete sequence
CAGL0L00407g	1	Putative adhesin with glycine and serine rich repeats
CAGL0L01089g	1	Similar to uniprot|P35187 Saccharomyces cerevisiae YMR190c SGS1 DNA helicase
CAGL0L01595r	1	Similar to uniprot|Q12680 Saccharomyces cerevisiae YDL171c GLT1 glutamate synthase

CAGL0L01639g	1	tRNA-Tyr
CAGL0L02585g	1	Ubiquitin carboxyl-terminal hydrolase (EC 3.4.19.12)
CAGL0L08624g	1	Similar to uniprot|P22035 Saccharomyces cerevisiae YKR099w BAS1 transcription factor
CAGL0L09955g	1	Similar to uniprot|P43583 Saccharomyces cerevisiae YFL007w
CAGL0L11792g	1	Polyadenylate-binding protein, cytoplasmic and nuclear
CAGL0M03465g	1	Strain CBS138 chromosome M complete sequence

Supplementary Table S7 Genes with SNP located in regulatory regions

Gene	Function
CAGL0C01133g	Similarities with uniprot|Q08294 Saccharomyces cerevisiae YOL155c
CAGL0E00187g	Strain CBS138 chromosome E complete sequence
CAGL0F00803g	Strain CBS138 chromosome F complete sequence
CAGL0F06061g	Strain CBS138 chromosome F complete sequence
CAGL0F09273g	Putative adhesin-like protein
CAGL0G02915g	Similar to uniprot|P53252 Saccharomyces cerevisiae YGR086c
CAGL0H09878g	Inorganic pyrophosphatase (EC 3.6.1.1) (Pyrophosphate phospho-hydrolase) (PPase)
CAGL0J11990g	Adhesin-like gene with internal repeats; predicted GPI-anchor; predicted pseudogene
CAGL0L01991g	Similar to uniprot|P36097 Saccharomyces cerevisiae YKL033w
CAGL0L03630g	Strain CBS138 chromosome L complete sequence
CAGL0L05236g	Malate dehydrogenase (EC 1.1.1.37)
CAGL0L05500g	Ribosome biogenesis protein ALB1
CAGL0L08624g	Similar to uniprot|P43583 Saccharomyces cerevisiae YFL007w
CAGL0M05797g	tRNA pseudouridine synthase (EC 5.4.99.-)
CAGL0M06325g	Strain CBS138 chromosome M complete sequence
CAGL0M09317g	Altered inheritance of mitochondria protein 24, mitochondrial
CAGL0M10329g	Similar to uniprot|P28778 Saccharomyces cerevisiae YKL003c MRP17
CAGL0M12496g	similar to YIL055C
CAGL0A01716g	Similar to uniprot|P53184 Saccharomyces cerevisiae YGL037c PNC1
CAGL0F05819g	Strain CBS138 chromosome F complete sequence
CAGL0K03443g	Similar to uniprot|Q04437 Saccharomyces cerevisiae YMR106c
CAGL0H02695g	Strain CBS138 chromosome H complete sequence
CAGL0A01738g	Strain CBS138 chromosome A complete sequence
CAGL0F05863g	Strain CBS138 chromosome F complete sequence
CAGL0K03421g	Similar to uniprot|P37012 Saccharomyces cerevisiae YMR105c Phosphoglucomutase 2

4. Identification and characterization of Adhesive proteins

Supplementary Table S8 Structure of Adhesive proteins

Gene	SP	low-comple	PA14	SHI	TTV	VSH	Flocculi	Hyphal_re	w	GPI anchor
		xity	domain	TT	VT	ITT	n	g_CWP	site	
**CGRM202019g59.t1**	√	√	√	--	--	--			321	D I L P D P P H T S F G P A P P P V T H T V
**CGRM202019g838.t1**	√	√	√	--	--	--			293	F V S T T N K E G V P I T G S T T I N G T R D Y L
**CGRM202019g3239.t1**	√	√	√	--	--	--			461	G D G K V V T T S T S T T Y S P P P V G T
**CGRM202019g4871.t1**	√	√	√	--	--	--			211	T D P E G V K R V D W N G Y I W R Y D S C
**CGRM202019g4671.t1**	--	√	√	--	--	--			283	S T T A S N G K P I T G T T T T T V I H T Y D P L
**CGRM202019g1098.t1**	√	√	√	--	--	--			368	G P G E G G S G S N P G T G E G G S G S
**CGRM202019g3116.t1**	√	√	--	1	--	1			423	Y Y G K T I T I T T T I P L Y G P A P P A V T
**CGRM202019g4605.t1**	--	√	--	--	--	--			182	Y H V L D Y F P V P Q D D K E H P Y G T
**CGRM202019g2400.t1**	--	√	--	--	--	--			908	H E S S S N S K T S I S L I V F I F G L D L V M F L C I M
**CGRM202019g60.t1**	√	√	--	--	--	--			568	V D L G N E V T E Y L V I S Y W T T T N E F G G L I
**CGRM202019g2401.t1**	√	√	--	1	1	--			625	S A V S T P S G Q D Y I I T T T G S D G K I E T D I
**CGRM202019g1979.t1**	√	√	--	--	--	--			201	A S S S T S A S S S T S A S S S S L Y V S A L S S V S A S
**CGRM202019g1062.t1**	√	√	--	--	--	--			540	A A S L N G L G S I G T L V T L L V L M L I N
**CGRM202019g836.t1**	√	√	--	--	--	--		√	449	T T T M T E L V S N I T T T D S N G K P T T I V
**CGRM202019g3233.t1**	√	√	--	--	--	--		√	430	E G W T G S V T N T R S T D F S T F T G
**CGRM202019g1978.t1**	√	√	--	--	1	--		√	476	T D K V T S D I T T V V T N S D G S S E T D
**CGRM202019g57.t1**	√	√	--	--	--	--		√		
**CGRM202019g1157.t1**	√	√	--	--	--	--			943	M G S S V I L P Y W T L A I F F I F H H I
**CGRM202019g1485.t1**	√	√	--	2	1	--		√	582	T I I I T T L G E P A T A S D Y T T V V T N S D G S V
**CGRM202019g4655.t1**	√	√	--	4	4	4			804	G A G S T F K I K F N T I L L S T S L T I L I L L G M A
**CGRM202019g3003.t1**	√	√	--	--	--	--		√	583	Y L T L T T M T P T D N P K V T P T N V V T T
**CGRM202019g2780.t1**	√	√	--	2	1	--		√	587	T T I K S S K E T D D Y T T V V T N A D G S V E T
**CGRM202019g3515.t1**	√	√	--	--	--	--			718	H G G V E H V N V G L F N F M L P F I I S V L L L
**CGRM202019g3514.t1**	√	√	--	--	--	--			850	G C G T V N H K G M I N I L L A L A L L V L L
**CGRM202019g3516.t1**	√	√	--	--	--	--			714	H G G V E K I N V G L F Q V L L P F V V S V L L L
**CGRM202019g3005.t1**	--	√	--	--	--	--			192	Y Y I A S D D T G E H V A T K T I T V K
**CGRM202019g1063.t1**	√	--	--	--	--	--			371	T M I T I C K K E D Y T T T I D K G N G D F E T
**CGRM202019g3484.t1**	√	√	--	--	--	--			418	A E T P E A D Y T T T I T S D G H T I T
**CGRM202019g3984.t1**	√	√	--	--	--	--			402	D D T G Q H I A T K T V T V K V E E P D Y T T T Y
**CGRM202019g835.t1**	√	√	√	--	--	--			358	H L T P T Y D Y T K T V T S D G H T I T E V V S
**CGRM202019g1436.t1**	--	√	√	--	--	--			338	S Q E Y S S S S H R L P S S S H A L I H S S S V
**CGRM202019g3117.t1**	√	√	√	--	--	--			335	S S S S S S S S S S S S S S S S P S V N P S S V N P S S V N P
**CGRM202019g3623.t1**	√	√	√	--	--	--			375	G D I T I T L T K P S G E A D Y T T T I T S D G H

**CGRM202019g3670.t1**	√	√	√	--	--	--			415	D G G M L V E G Q C D Y Q C K E A G G I L V G
**CGRM202019g2727.t1**	√	√	√	--	--	--			437	P S P S P S P S P S P S P S P S P S X X X X A
**CGRM202019g61.t1**	√	√	√	--	--	--			854	G G S T F L K A S I S Y L S L T E L I I G V I L L C
**CGRM202019g1158.t1**	√	√	√	--	--	--			588	A G S N E T S I N V P F S S S T E S N A S T S S T
**CGRM202019g2779.t1**	√	√	--	--	--	--			484	T A S S V S T F N T I I P T A S T T Y V P G S V S T
**CGRM202019g4656.t1**	√	√	√	--	--	--		√	319	K N T C Y Y P K P E D P K P E D P K P E
**CGRM202019g3240.t1**	√	√	√	--	--	--		√	396	G S V I T T E A T K T Y S P P P V T I T A T T A A
**CGRM202019g4870.t1**	√	√	√	--	--	--			342	V N P S S V N P S S V N P S S K P A D P S P A
**CGRM202019g2330.t1**	--	√	√	1	--	1			305	Y A E N G P G L Y T S F G P A P P V E T H T V V
**CGRM202019g1444.t1**	√	√	--	--	--	--			548	D Y T T T I D K G N G D F E T D L V S H
**CGRM202019g4321.t1**	√	√	--	--	--	--		√	448	Y D P A P G P E T K I V D L G N E V T E Y D V I S Y Y T
**CGRM202019g3485.T1**	--	√	--	--	--	--	--	--	286	A A T I K A T S N T A I L V L I I L F F L G L T
**CGRM202019g841.t1**	√	√	--	--	--	--	--	√	323	G Q H E N H D S G S G S T P G E G S G S G
**CGRM202019g1337.t1**	√	√	--	--	--	--	--	√	324	E D I P P I E P S S V K P S S M N P S S V N P S
**CGRM202019g3454.t1**	--	√	--	--	--	--			296	T F R G S A V M L H V Y G I E L V L P V L A A L L F I
**CGRM202019g3674.t1**	√	√	√	--	--	--	√	--	390	C D K S C X X X X A R T R A R T R T R A

Supplementary Table S9 Twelve changed pseudogenes

Pseudogenes	Distribution	Pseudogenes	Distribution
CAGL0B00110g	dispersed	CAGL0D06732g	concentrated
CAGL0B05093g	dispersed	CAGL0E00110g	concentrated
CAGL0A00110g	dispersed	CAGL0G00110g	concentrated
CAGL0J11990g	dispersed	CAGL0I00110g	concentrated
CAGL0F00110g	dispersed	CAGL0J00110g	concentrated
CAGL0D00110g	concentrated	CAGL0C01067g	concentrated
